# Impact of various detergent-based immersion and perfusion decellularization strategies on the novel caprine pancreas derived extracellular matrix scaffold

**DOI:** 10.3389/fbioe.2023.1253804

**Published:** 2023-09-18

**Authors:** Garima Singh, Sanghamitra Satpathi, Bora Venu Gopala Reddy, Manish Kumar Singh, Samchita Sarangi, Prativa Kumari Behera, Bismita Nayak

**Affiliations:** ^1^ Immunology and Molecular Medicine Laboratory, Department of Life Science, National Institute of Technology, Rourkela, India; ^2^ Department of Pathology, Hi-Tech Medical College and Hospital, Rourkela, India; ^3^ Department of Histopathology, Ispat General Hospital, Rourkela, India

**Keywords:** caprine pancreas, extracellular matrix (ECM), decellularization, 3D scaffold, tissue engineering, collagen, sulfated glycosaminoglycans (sGAG)

## Abstract

Limited availability of the organs donors has facilitated the establishment of xenogeneic organ sources for transplantation. Numerous studies have decellularized several organs and assessed their implantability in order to provide such organs. Among all the decellularized organs studies for xenotransplantation, the pancreas has garnered very limited amount of research. The presently offered alternatives for pancreas transplantation are unable to liberate patients from donor dependence. The rat and mice pancreas are not of an accurate size for transplantation but can only be used for *in-vitro* studies mimicking *in-vivo* immune response in humans, while the porcine pancreas can cause zoonotic diseases as it carries porcine endogenous retrovirus (PERV- A/B/C). Therefore, we propose caprine pancreas as a substitute for these organs, which not only reduces donor dependence but also poses no risk of zoonosis. Upon decellularization the extracellular matrix (ECM) of different tissues responds differently to the detergents used for decellularization at physical and physiological level; this necessitates a comprehensive analysis of each tissue independently. This study investigates the impact of decellularization by ionic (SDS and SDC), non-ionic (Triton X-100 and Tween-20), and zwitterionic detergents (CHAPS). All these five detergents have been used to decellularize caprine pancreas via immersion (ID) and perfusion (PD) set-up. In this study, an extensive comparison of these two configurations (ID and PD) with regard to each detergent has been conducted. The final obtained scaffold with each set-up has been evaluated for the left-over cytosolic content, ECM components like sGAG, collagen, and fibronectin were estimated via Prussian blue and Immunohistochemical staining respectively, and finally for the tensile strength and antimicrobial activity. All the detergents performed consistently superior in PD than in ID. Conclusively, PD with SDS, SDC, and TX-100 successfully decellularizes caprine pancreatic tissue while retaining ECM architecture and mechanical properties. This research demonstrates the viability of caprine pancreatic tissue as a substitute scaffold for porcine organs and provides optimal decellularization protocol for this xenogeneic tissue. This research aims to establish a foundation for further investigations into potential regenerative strategies using this ECM in combination with other factors.

## Graphical Abstract

**Figure:**
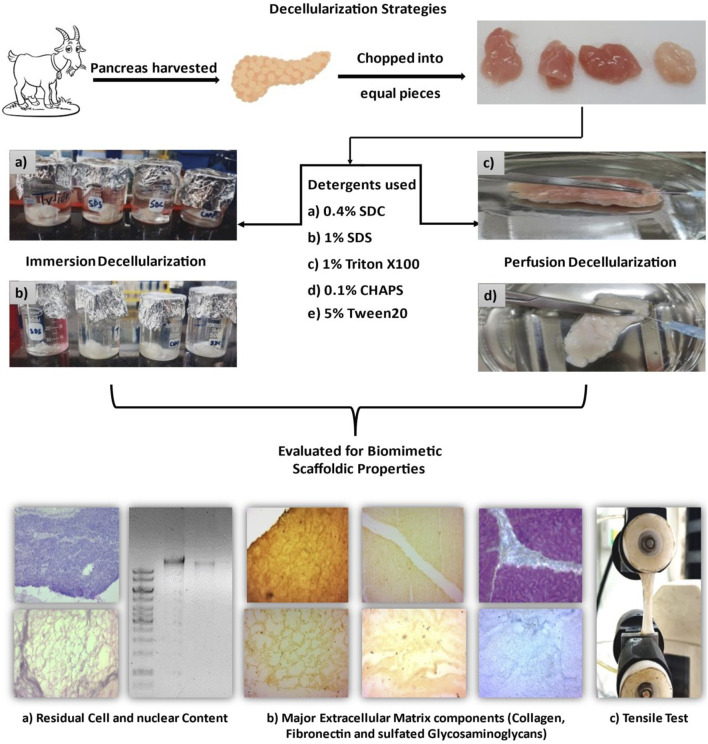

## 1 Introduction

Tissue engineering is a multidisciplinary science that combines engineering, biology, and medical concepts to construct viable live tissues and organs. It combines the use of cells, biomaterials, and biological factors to construct 3D frameworks that imitate the function and structure of native tissues. Without triggering the recipient’s immune system upon transplantation (Freed and Guilak, 2007). The principal objective of tissue engineering is to overcome the shortcomings of the existing biomaterial implants by developing bioartificial organs that are immunologically compatible. This would lead to a long-term solution for the affected organ without repeated therapies, making it an economically viable therapeutic technique (Yesmin et al., 2017). To address potential immune reactions, strategies can be employed to modify the ECM and optimize its compatibility with the recipient’s immune system. The success of using decellularized organs as transplantable grafts without extensive immunosuppression relies on refining the decellularization process, optimizing the ECM for host cell infiltration, and potentially combining this approach with recipient-specific cell-based therapies (Tan et al., 2021). These multidisciplinary strategies can help to mitigate immune rejection concerns and improve patient outcomes. Throughout the development of these artificial organs, various aspects, such as biocompatibility, oxygen and nutrient availability, immunological rejection, etc., must be taken into account. As cells are accustomed to proliferating in a 2D environment, the matrix of these decellularized organs provides external cues to direct their proliferation into a 3D environment (Zhu et al., 2019). In addition to its usage as an organ replacement scaffold, such decellularized matrix has several alternate applications, including tubes, sheets, hydrogel, powder, bio-printing inks (Kort-Mascort et al., 2021), and even cartilage regeneration (Chakraborty et al., 2023). The most sought-after approaches in the field of tissue engineering are regenerative ones. Regenerative approaches include harvesting of a potential auto/allo/xenogeneic organ, removal of the native cells from this organ (decellularization), recellularization of this tissue/organ, and screening its *in-vitro* and *in-vivo* functionality to ascertain its viability as bio-scaffold for transplantation (Badylak et al., 2011). This method leads to constructing a 3D scaffold biomaterial integrated with cells and/or factors, which ultimately becomes a promising tool for restoring or replacing the function of a damaged organ or tissue (Liu et al., 2007). Recently, ECM derived from decellularized tissue has been adopted as a promising strategy to enable 3D tissue formation in a variety of porcine organs such as cartilage (Shen et al., 2020), urethra (Simões et al., 2017), lumbar spine (Wu et al., 2017), heart (Zhou et al., 2010), liver and kidney (Wang et al., 2015), and a few bovine organs like ovaries (Nikniaz et al., 2021), spine (Yu et al., 2020), trabecular bone (Shridhar et al., 2019), liver (Ergun et al., 2022) and lungs (Kuşoğlu et al., 2023). These decellularized tissues have successfully retained the ultrastructure as well as organ-specific microenvironmental elements, such as natural ECM proteins and growth factors, which are responsible for cell adhesion and proliferation. When intended for use in tissue engineering, these tissues must adhere to the stringent standards of biocompatibility, immunogenicity, and mechanical stability (Radulescu et al., 2022). Due to the parameters which are specific to individual tissues, including tissue thickness, size, morphology, as well as cell and matrix density constraints, preferred decellularization techniques differ across tissues and organs (Shen et al., 2020). Removing cellular remnants is crucial in this context; otherwise, post-transplantation, inflammation, fibrosis, and scar tissue accumulation may develop (Mao et al., 2017). These decellularized matrices are anticipated to maintain a balance between the biodegradation triggered by the host body and the host tissue formation. Therefore, it is important to carefully tailor the decellularization technique in terms of detergent type, period of exposure, mode of decellularization, and final ECM characteristics. Among the decellularized organs, porcine organs are widely researched because of their immunologic compatibility with humans (Ibrahim et al., 2006). Nevertheless, porcine organs would always be capable of harboring PERV (porcine endogenous retrovirus), which can induce zoonotic infections following xenotransplantation (Kim et al., 2016). A novel alternative to porine organs can be the goat (caprine) pancreas which has been documented to share physiological and structural similarities with the human pancreas (Bhuyan and Saikia, 2016). Besides these similarities, the caprine pancreas is usually discarded by the meat industry/butchers; thus, its procurement will not incur additional costs. This organ can serve as a viable xenogeneic source as it has immense potential to replace the porcine organs, thereby reducing the risk of zoonotic diseases, which requires comprehensive research for each organ separately. Therefore, this work aims to design a decellularization approach unique to the caprine pancreas. Immersion in a detergent solution with mechanical stirring is one of the most popular methods for hydrolyzing cell membranes and detaching DNA along with other cellular components from the ECM (Hazwani et al., 2019). The other method of decellularization involves perfusing the tissue with detergents; this has also proven effective in achieving the ideal scaffolds (Garreta et al., 2017).

Immersion decellularization (ID) and perfusion decellularization were therefore selected as the two decellularization techniques (PD) to examine their efficacy for decellularizing caprine pancreatic tissue. The final outcome of these techniques is fully reliant on the tendency of the detergents to permeate into the tissue. Priority is given to the efficacy of the decellularization approach in order to guarantee the quality of the final matrix. This work will help to determine the finest decellularization strategy for the caprine pancreas while retaining its native ECM architecture and minimum loss to elasticity. The native tissue has superior structural complexity of ECM, mainly owing to fibrous proteins (collagen, fibronectin, tenascin, elastin, laminin), sulfated glycosaminoglycans (sGAG) (hyaluronic acid, chondroitin sulfate, dermatan sulfate, keratan sulfate, and heparan sulfate) (Kim, 2017). After decellularization, the expected molecules to persist are fibrous proteins and sGAG; since the fibrous proteins are crucial for the biocompatibility of cells as well as the scaffold’s elasticity (Meyer, 2019). On the other hand, sGAG interacts with other ECM components, including fibrous proteins such as collagen and elastin, signaling molecules, growth factors, and enzymes, to regulate cell activity, tissue development, and repair processes (Mohammadi et al., 2017). Several structural characteristics, such as charge distribution, size and rigidity of hydrophilic and hydrophobic groups, and peculiar interconnections between detergents and proteins, determine the propensity of detergents to denature proteins in the ECM (Privé, 2007). It is rational and foreseeable that using such detergents will damage the ECM during decellularization; nevertheless, this disruption should not render the scaffold entirely devoid of biocompatibility and mechanical traits. Since the caprine pancreas has not been largely investigated yet, this study will assist in further analyzing and developing an optimal strategy for recellularization and subsequent *in-vivo* immunological study of the same so it can be implemented for a broad range of biomimetic applications.

## 2 Materials and methods

### 2.1 Harvesting and decellularization of the Caprine pancreas

The goat pancreas was purchased from the local butcher’s shop immediately after the goat was slaughtered. The pancreas was excised aseptically and submerged instantly in ice-cold 1XPBS. It was carried in an ice box from the butcher shop to the laboratory with utmost safety precautions. After disinfecting the pancreas with 0.1% peracetic acid and PBS, it was dissected into small, equal pieces measuring 15 mm*15 mm each. For subsequent performance assessment, each tissue segment was subjected to a distinct mode of decellularization, as listed in Table 1.

**TABLE 1:** Brief description of the protocols followed for the mode of decellularization of the caprine pancreas using various concentrations of the detergents.

Groups	Decellularization strategy	SDC	SDS	Triton X-100	CHAPS	Tween-20
Group 1 (5 Pancreatic Tissue sections)	Immersion Decellularization (ID): Tissue sections were submerged in the 5 specified concentrations of detergents and stored in an airtight jar until the pancreas turned pure white. The tissue underwent a 3-h detergent cycle followed by a 1XPBS rinse at the completion of each cycle. Tissues were finally washed with 1XPBS and 0.1% Peracetic acid for 48 h, with 4 changes of each solution	0.4% (w/v)	1% (w/v)	1% (v/v)	0.1% (w/v)	5% (v/v)
Group 2 (5 Pancreatic Tissue sections)	Perfusion Decellularization (PD): A 16G needle was inserted into the midsection of pancreatic tissue. The sliced section was perfused with the five different indicated detergents using a gravity-driven apparatus. These sections were perfused with detergents until they turned white as opposed to their usual pale pink color. At the end of decellularization cycle, the tissue was perfused with 1XPBS and 0.1% Peracetic acid for 48 h, with four changes of each					

### 2.2 Decellularization cycle progress and duration

Alteration in the color and density of the detergent effluent indicated that cells were detaching from ECM and draining. These native cells made the detergent efflux partially turbid. This detergent effluent was collected at every fourth hour of decellularization in order to assess the cellular debris in the discharge. The absorbance of the collected effluent was measured at 280 nm using a Shimadzu UV–Vis 1800 Spectrophotometer to estimate turbidity. The absorbance curve *versus* time was graphed to determine the timeframe where the highest cellular clearance occurred.

**Note:** We have decided not to agitate the tissue in ID as it might lead to additional ECM destruction, and the primary goal of this study is to evaluate the impact of different detergents on the final ECM without using any other external forces. For the same reason, no additional decellularization chemicals, such as protease inhibitors or DNase, are included in the PD. The same reason lies behind not including any other decellularization additives like protease inhibitors or DNase in the PD also.

### 2.3 Tissue fixation and block preparation

A tissue section measuring 5 mm*5 mm*5 mm was fixed in 10% neutral buffered formalin for 48 h at RT. Using a Leica-RM2235 microtome, paraffin wax blocks of tissue were sectioned at a thickness of 5 µm. The sections were subsequently transferred to positively charged microscope slides (BioMarq- SL001-50) and stained.

### 2.4 Nuclear material estimation and quantification

DNA was isolated from each sample (*n* = 6 of each detergent from ID and PD) using HiPurA^®^ Mammalian Genomic DNA purification kit (MB506) according to the manufacturer’s instructions. To quantify the DNA, absorbance was taken (as ratio of 260/280 nm) using Eppendorf BioPhotometer^®^D30. The extracted DNA was subsequently measured as ng/g of dry tissue mass. DNA was evaluated qualitatively by running the samples on 1% w/v agarose gel at 100 V until the dye reached 3/4th of the distance from wells. Using a DNA ladder (NEX-GEN, Puregene PG010-500DI-NV) ranging from 250 to 10,000 bp, the amount of DNA present in the obtained tissue sections was estimated. For DAPI staining, the sections were dewaxed, rehydrated, and stained with DAPI, followed by PBS washing to eliminate the non-specific staining. The slides were mounted with an antifade reagent [p-phenylenediamine in 90% glycerol (v/v) in 0.1 M PBS], and sections were viewed at 460 nm using Leica TCS SP8 confocal laser scanning microscope (Robertson et al., 2014).

### 2.5 H&E staining

H&E staining was performed to measure the efficacy of each detergent at decellularizing the tissue. The tissue slides were rehydrated and stained with H&E. Following successive dehydration; the slides were fixed with DPX and examined under a Leica-DM2500 light microscope. Leica LAS EZ imaging software was used to acquire all the micrographs.

### 2.6 sGAG imaging

Following deparaffinization and rehydration, the slides were incubated for 12 min in 12% glacial acetic acid (GLAA). The slides were then immersed in Prussian blue solution for 1 hour. The slides were then rinsed twice for 3 min with 12% GLAA. The mixture of 20% HCl and 10% potassium ferrocyanide was left in contact with the slides for 30 min. Later, these slides were counterstained with nuclear fast red for 5 min. The slides were thereafter dehydrated, cleared, and mounted with DPX for imaging.

### 2.7 sGAG Content estimation

The decellularized tissue sections from ID and PD (*n* = 3 each) were lyophilized, and 20 mg dried tissue was digested Collagenase type I (from *Clostridium histolytica*) in PBS for 2 days at RT. Using a DMMB assay (Zheng and Levenston, 2015), the sGAG content of this solubilized tissue was determined. Chondroitin sulfate (from shark cartilage) concentrations ranging from 1 to 50 μg/mL have been used to plot the standard curve. A 200 µL concoction of digested tissue solution, PBS, and DMMB reagent were plated in each well of a 96-well plate to determine the amount of sGAG in decellularized tissues. The absorbance was measured at 590 nm using a ThermoScientific^®^ MULTISKAN SkyHigh plate reader. The sGAG concentration was calculated as µg/mg of dry tissue mass after adjusting the absorbance to the standard curve.

### 2.8 IHC staining

Crucial ECM proteins collagen and fibronectin were measured in all the sections of DT of PD and compared to NT. The primary antibody used for collagen was mammalian collagen I (1:100, COL1A1G3, Santa Cruz Biotechnology), and for fibronectin was mammalian fibronectin (1:100, Fibronectin-EP5, Santa Cruz Biotechnology). The secondary antibody used was anti-mouse IgGκ (1:50, BP-HRP, Santa Cruz Biotechnology) coupled to Horseradish peroxidase. Protease-induced epitope retrieval was carried out in accordance with literature recommendations for ECM (Rickelt and Hynes, 2018). The droplets of primary antibodies for each protein were added and incubated overnight at 4°C in a humid chamber. The following day, secondary antibody drops were added to each sample and incubated for 2 h in a humid environment. Slides were washed with 1XTBST buffer, dehydrated with ethanol, cleared in xylene, and mounted using DPX to be viewed under a microscope. Note: According to the quantification, ID tissues lacked considerable quantities of collagen; hence they were not tested for IHC imaging.

### 2.9 Collagen quantification

The standard Hydroxyproline method (Cissell et al., 2017) was used to assess the residual quantity of collagen in DT. The DT samples from both ID and PD (*n* = 3 each) were lyophilized, and 20 mg of this dried tissue was digested with papain at 60°C overnight. Collagen (SRL-90443, ex. marine fish) concentrations ranging from 0.5 to 5 μg/mL were used to yield the standard curve. Using the ThermoScientific^®^ MULTISKAN SkyHigh plate reader, the absorbance was measured at 560 nm. Using the standard curve, the resultant collagen concentration was normalized to the sample’s dry weight.

### 2.10 SEM imaging

All the DT samples from ID and PD were primarily fixed in a concoction of 2.5% glutaraldehyde and 2% paraformaldehyde for 8 h at 4°C. Secondary fixation was achieved with 0.2% osmium tetroxide for 2 h in the dark at RT. Following fixation, the samples were washed 3 times for 15 min each with distilled water, proceeded by dehydration with gradually increasing ethanol concentration. Subsequently, the JEOL JFC-1600 auto fine sputter coater was used to sputter-coat platinum onto the critical point-dried tissue samples. Under a JEOL JSM-6480LV Scanning Electron Microscope, the final specimen was observed.

### 2.11 Swelling index study

The lyophilized DT samples (*n* = 3 for each) from ID and PD were weighed and submerged in 1XPBS at 37°C for 24 h to determine the scaffold’s swelling index. The tissue segments were weighed every 2 h, and the maximal weight gain was deemed the final weight or maximum swelling for each sample. The swelling index was determined using the formula: W_s_ = {(W_f_ − W_0_)/W_0_} × 100, where W_s_ = swelling index, W_0_ = initial weight, W_f_ = final maximum weight.

### 2.12 Biodegradability analysis

Both native and DT samples (*n* = 6) were immersed in 1XPBS (pH7.4) containing 0.2% collagenase (from *Clostridium histolyticum*, Sigma-Aldrich-SCR103) and incubated in a 37°C shaker incubator for biodegradation pattern analysis. The initial weight was determined by weighing all the samples before exposing them to the enzymatic solution, and subsequent weights were recorded every 4 h for 24 h. The biodegradability percentage was calculated as: D = {(W_0_-W_t_)/W_t_} *100, where D = weight loss percentage, W_0_ = Initial weight of DT, W_t_ = weight of degraded tissue at time t (*t* = timepoint at every 4 h).

### 2.13 Tensile property test

The tensile modulus of a tissue reflects the extent to which it can return to its former shape after being expanded. NT (*n* = 6) and DT (*n* = 6 each of ID and PD) tissue samples immersed in 1XPBS were subjected to uniaxial tensile testing to determine mechanical strength to ensure no sharp decline in elasticity. The tissue samples were trimmed into dog-bone-shaped rectangular pieces of equal dimensions with length = 20 mm, width = 12 mm, and thickness = 5 mm. The clamp grip of the electromechanical machine Instron E1000 ElectropulsTM at RT was used to secure both wide ends of the tissue. It was then stretched at a 5 mm/min cross-head speed with a growing pressure of 1–20 N.

### 2.14 Cytocompatibility assay

The cytotoxicity of the resulting DT (*n* = 3 of each ID and PD) was investigated against MIN-6 (RPMI-1640) cells for 24, 48, and 72 h. For this analysis, the decellularized segments were immersed in culture media RPMI-1640 for 72 h at 37°C and 5% CO_**2**_. Thereafter, increasing concentrations ranging from 10 to 100 μL (in triplicates) of this DT-immersed media were added to each well of a 96-well plate containing MIN6 cells. This increasing concentration of DT-soaked media was incubated with MTT, and absorbance was measured at 590 nm with ThermoScientific^®^ MULTISKAN SkyHigh plate reader.

### 2.15 Antimicrobial assay

The antibacterial activity of decellularized matrices was evaluated against Gram-positive *S. aureus* using standard procedures. MH broth and MH agar were prepared following the manufacturer’s guidelines and verified for sterility by overnight incubation at 37°C. *S. aureus* was streaked on MH agar plates, and isolated colonies in the lag phase of growth (two to three colonies) were transferred to vials containing 10 mL of MH broth. The cultures were then incubated at 37°C overnight in a shaker until they reached an optical density of 0.1 at 570 nm. Upon reaching the desired density, the bacterial cultures were harvested, diluted to 105 CFU/mL, and 100 μL of the bacterial suspension was added to a 15 mL centrifuge tube. Simultaneously, 10 gm of decellularized matrix was suspended in 10 mL of 1X PBS containing collagenase type I (2 mg/mL). The matrix was incubated in a shaker at 37°C until complete degradation occurred, with no visible tissue remains. Subsequently, 100 μL of the degraded tissue was added to the 15 mL centrifuge tube containing bacterial suspension. To act as a positive control, tetracycline was prepared as a stock solution (10 mg/mL) in 50% methanol. MH broth without the matrix served as the negative control. To monitor the antibacterial activity, all the tubes were kept at 37°C incubator, and absorbance was measured at regular intervals at 570 nm using Shimadzu UV–Vis 1800 Spectrophotometer. All assays were performed in triplicate to ensure accuracy and reduce errors in the experimental procedure.

### 2.16 Statistical analysis

ImageJ software was used to process and scale the photos. The data are presented as the mean and standard error of means (±SEM). All statistical tests were conducted using SPSS (version 24 for Windows; IBM^®^). Statistical evaluation was conducted with one-way ANOVA and the Tukey test for multiple comparisons with *post hoc* HSD (honestly significant difference) analysis. All data values for *n* = 3–6 was depicted as mean ± SEM, taking a 95% confidence interval. A *p*-value less than 0.05 indicated a statistically significant outcome. All the *p*-values are with reference to the native tissue unless stated otherwise.

## 3 Results and discussion

### 3.1 Decellularization cycle progress and duration

**FIGURE 1:**
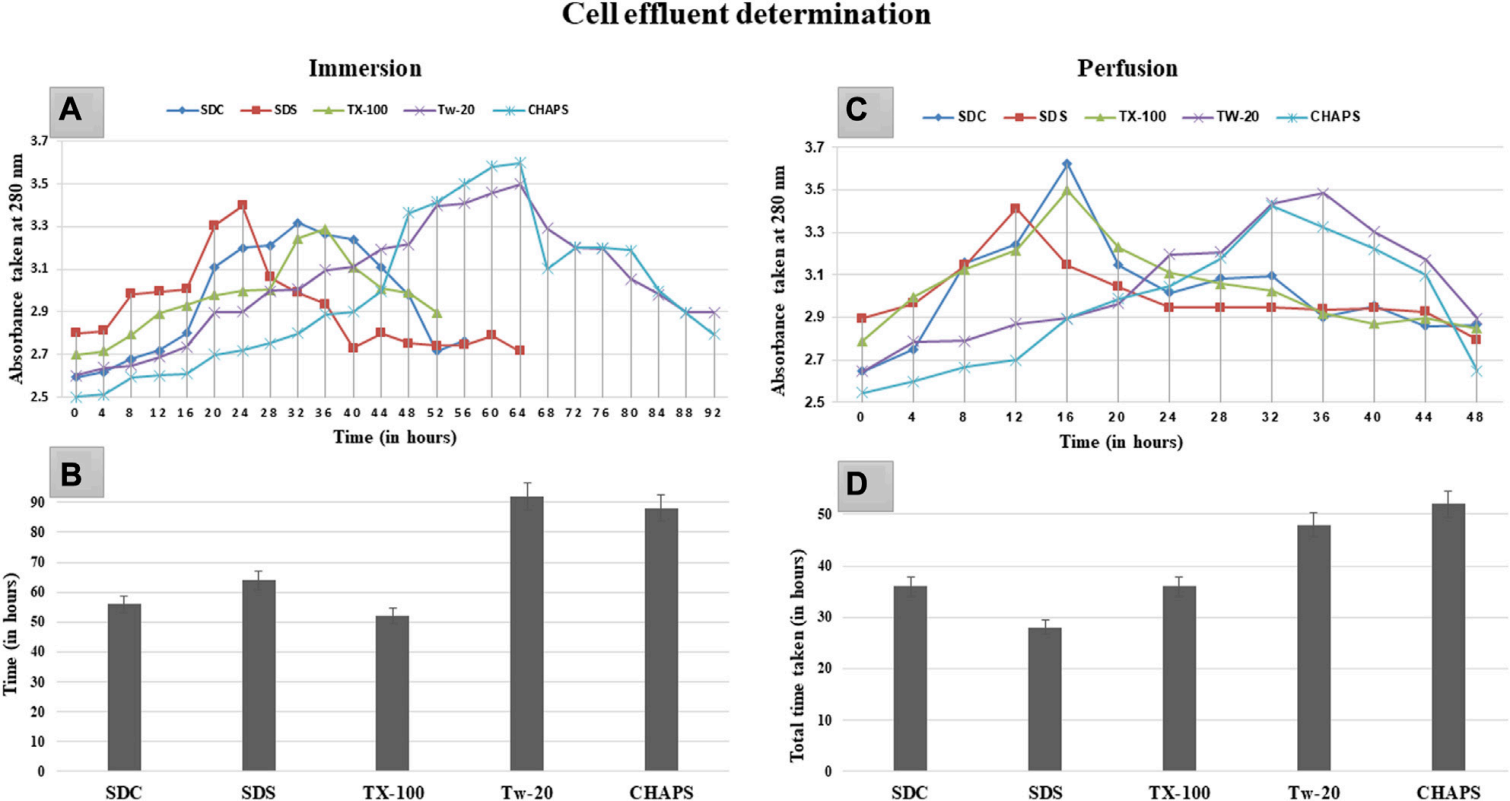
After 20 h in ID **(A)**, SDS and SDC indicated turbidity, while TX-100 displayed turbidity after 28 h. However, CHAPS and Tw-20 were the last to exhibit this turbidity associated with cell effluent, after 48 h. The cumulative time consumed by SDS, SDC, and TX-100 **(B)** to complete the decellularization was almost similar in ID, i.e., between 52 and 65 h, whereas TW-20 and CHAPS took considerably longer duration to decellularize. For PD, **(C)** turbidity appearance was reasonably early for all the detergents, especially TX-100, SDC, and SDS, while Tw-20 and CHAPS were still found to be the slowest among all. Since the turbidity corresponding to the cell effluent was detected in PD earlier than in ID, the time required for complete decellularization in PD **(D)** was also considerably shorter.

Prolonged exposure to the detergent can induce deterioration of the extracellular matrix (ECM) in addition to the removal of cellular material (White et al., 2017). Therefore, the ideal detergent must not only accomplish decellularization without harming the ECM but also do so in the shortest duration of time possible. Upon complete cell removal, the ECM of the DT samples was translucent and white. The rate of decellularization was established by monitoring the effluent turbidity, as maximum cell elimination corresponded to the absorption maxima at that time. In ID, the sharp rise in graph peak was observed first with SDC and SDS at 20 h of the time stamp (Figures 1A). In comparison, TX-100 took a substantially longer time (32 h) to attain this peak. In contradiction to the other three detergents in the ID, CHAPS, and Tw-20 required a notably extended amount of time, with the effluent absorbance peak arising at 64 h. These peaks essentially exhibited a significant level of cellular clearance at the given time; however, total decellularization took additional time, as depicted by the endpoint of each detergent curve (Figures 1B). Tw-20 and CHAPS decellularized the same area of the caprine pancreas in nearly 80 h, the longest of the five detergents evaluated in ID. The remaining detergents decellularized the same sample in 50–65 h, proving their efficacy. Each detergent’s performance profile regarding the time taken to decellularize improved in PD. One plausible explanation is that more tissue surface area was exposed to the detergent, resulting in a shorter decellularization period for these detergents in PD (Figures 1D). While in ID, this tissue surface exposure to detergent was remarkably low, leading to delayed cell clearance. Noteworthy in ID effluent was the formation of a plateau rather than a peak, suggesting a gradual loss of core cells over time. The peak in the effluent was only found in PD (Figures 1C), showing that the effluent leaving the tissue contains eliminated inner core area cells. This sudden spike was found amiss in the ID as it has shown a gradual decrease in cellular content, giving rise to the plateau region instead of a spike. The most probable reason for this plateau in the ID curve is that the cells from the outer borders of the tissue were removed first, followed by the more densely populated core cells, resulting in the effluent having a continuous peak, i.e., a plateau. We found that Tw-20 was not very impactful at decellularizing the caprine pancreas, and a similar result was also obtained upon decellularization of the equine tendon (Aeberhard et al., 2020). But when used in concoction with other detergents, it has shown increased decellularization efficacy in the case of ovine aorta (Heidarzadeh et al., 2021).

### 3.2 Nuclear material estimation and quantification after decellularization

**FIGURE 2:**
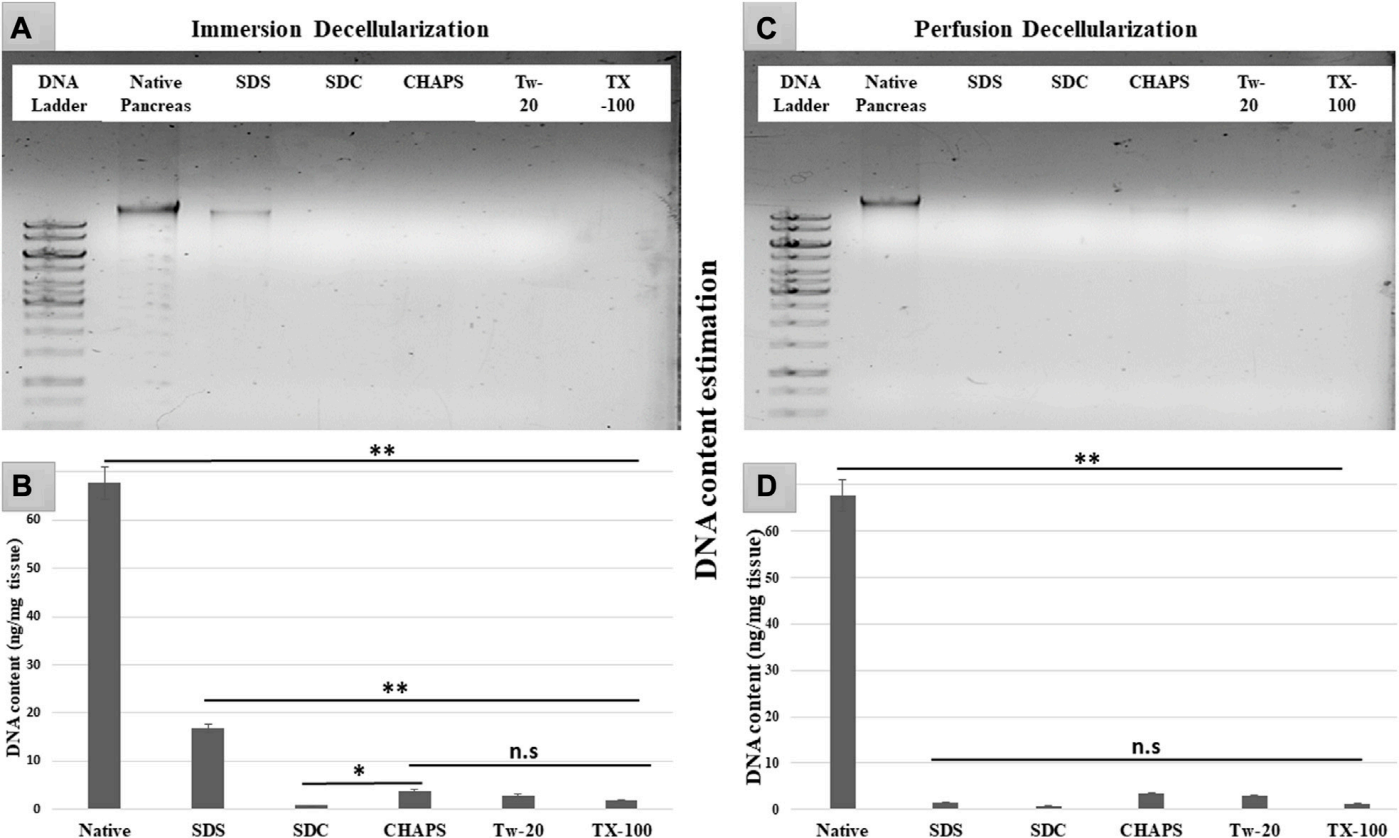
DNA imaging of ID has indicated that SDS was unable to eliminate the genetic material altogether resulting in the appearance of a DNA band **(A)** in SDS lane. During DNA quantification of ID **(B)**, native tissue was found to have a substantial difference with all the decellularized counterparts (**p < 0.001). Although SDS DNA content **(B)** is markedly lower as compared to the native, it also has shown a considerably higher DNA amount (**p < 0.001) than other detergents. In contrast, CHAPS **(B)** varied significantly from SDC (*p < 0.05) but showed no discernible difference (*p* > 0.05) from rest of the detergents used in ID. While in PD, all the detergents have gotten rid of the DNA effectively **(D)** (**p < 0.001), showing no significant variance (*p* > 0.05) from each other.

**FIGURE 3:**
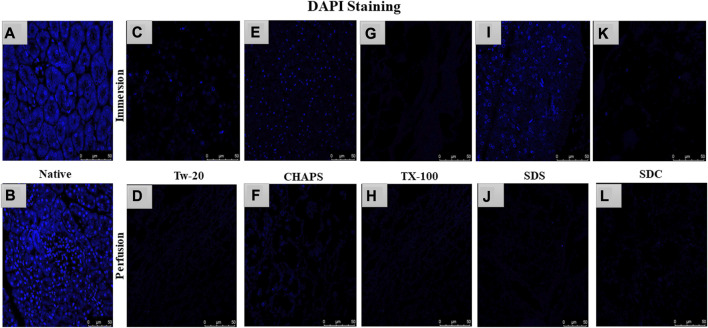
DAPI staining concluded that native tissue had genetic material present **(A, B)**, while PD set-up successfully eliminated the DNA. In PD only, Tw-20 **(D)** and CHAPS **(F)** has residual cytoplasm but not DNA. The ID set-up was not able to remove the DNA completely hence the staining was evident in Tw-20 **(C)**, CHAPS **(E)** and SDS **(I)**, while TX-100 has shown better DNA removal efficacy even in ID **(G)**.

After removing the cellular content, the nuclear material was examined to ensure that none was left behind. The leftover nuclear material is potentially harmful as a putative immunogen for transmitting zoonotic diseases. In scaffolds, a DNA concentration of more than 20 ng/mL is known to be detrimental (Crapo et al., 2011). It was ascertained by DNA quantification and imaging that none of the detergents retained this quantity. With the exception of SDS, all other detergents in ID eliminated DNA (Figures 2A). SDS is primarily used to denature proteins; however, its low doses have been proven to adequately remove the native cells while releasing DNA-protein couplings and keeping the DNA intact (Zhitkovich and Costa, 1992). SDS does not interact with DNA, and in ID, it was presumably unable to remove the native cells in the tissue core, which led to the formation of this DNA band (Figures 2A). The residual amount of DNA left behind by SDS (17.43 ± 1.18 ng/mg) differed significantly from that in native tissue (67.74 ± 0.69 ng/mg). Therefore, it cannot qualify as immunogenic, as the immunogen threshold of DNA is 20 ng/mL, which it did not surpass. In agreement with our DNA quantification data, Poornejad et al. have also reported that SDS alone was insufficient to eliminate the cellular/nuclear remnant (Poornejad et al., 2016). Its concentration can be increased to get rid of the DNA completely, but that would lead to degradation of the ECM matrisome, resulting in microstructural changes that ultimately reduce the biomechanical integrity of the ECM (Simsa et al., 2018). Since DNA fragments cling to the ECM after being discharged from the cell nucleus in the ID set-up, an additional washing step must be performed to remove it. DAPI staining was conducted to further validate this quantification of residual genetic material. Since DAPI is an indicator of DNA presence, hence it was typical for native tissue to radiate DAPI fluorescence throughout the entire section (Figures 3A, B), while no such fluorescence was recorded with Tw-20, TX-100, SDS, and SDC in PD (Figures 3D, F, H, J, L). On the other hand, ID set-up clearly failed to remove the DNA as seen in Tw-20, CHAPS, and SDS (Figures 3C, E, I), and only TX-100 and SDC were capable of doing so even in ID (Figures 3G, K). Other detergents in ID have substantially reduced DNA content (Figure 2B) as SDC, CHAPS, Tw-20, and TX-100 had 0.83 ± 0.31 ng/mg, 3.86 ± 0.32 ng/mg, 2.95 ± 0.11 ng/mg and 1.99 ± 0.37 ng/mg of DNA respectively. Among these, SDS has a lower content of residual DNA than native but considerably higher than other detergents (*p* < 0.0001). SDC only differed significantly from CHAPS (*p* = 0.04). CHAPS, Tw-20, and TX-100 have not revealed any major statistical variations in ID (*p* > 0.325). In PD, a weak DNA band was obtained only in the CHAPS lane apart from the native (Figure 2C), showing a slightly higher DNA content by CHAPS compared to its counterpart in ID. CHAPS is more suited for decellularizing only thin tissues due to its lower permeating capabilities, limiting its capacity to extract nuclear DNA (Marin-Tapia et al., 2021). Identical to SDS in ID, CHAPS DNA content in PD cannot be termed immunogenic because it does not outpace the immunogenicity threshold. SDS has performed better in PD, with a DNA content of 1.48 ± 0.31 ng/mg (Figure 2D), while other detergents SDC, CHAPS, Tw-20, and TX-100 had 0.82 ± 0.13 ng/mg, 3.38 ± 0.38 ng/mg, 2.87 ± 0.67 ng/mg and 1.17 ± 0.42 ng/mg of DNA content left respectively. All the detergents have shown highly significant differences (*p* < 0.0001) from native in PD, although no significant variation among each other (*p* > 0.998). This study reveals that PD is a superior method for decellulrizing the caprine pancreas by using any mentioned detergents in the given concentration, to remove the DNA, which plays a critical role in scaffold reseeding for *in-vivo* application.

### 3.3 H&E staining

**FIGURE 4:**
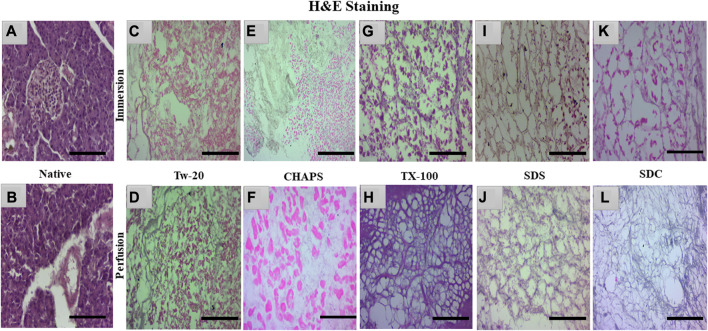
Native tissue of the caprine pancreas **(A, B)** stained with H&E displayed densely populated islets and acinar cells. In both ID and PD, Tw-20 did not perform adequately; this detergent left behind residual cytoplasm (**(C)**, pink distorted patches), lacking a defined ECM perimeter (**(D)**, distorted black fibrils). While CHAPS had residual cytoplasm (**(E)**, bright pink spots) in ID, disrupted ECM strands were observed in PD (**(F)**, pale blue interconnected strands). TX-100 had a large number of leftover cells attached to the ECM (**(G)**, dark pink blots) in ID, whereas, in PD, the ECM network of this detergent was discrete along with well-defined borders (**(H)**, purple mesh-like structure). SDS had an intact ECM network (**(I)**, brown interwoven network) in ID, but remnant cytoplasm was also spotted (**(I)**, pink blots). In contrast, the ECM network appeared undamaged in PD of SDS (**(J)**, faded purple interconnected network). SDC performed well in PD, yielding clear ECM devoid of cellular debris (**(L)**, faded purple mesh-like structure); however, this residue was detected in its ID (**(K)**, pink blots). (Scale bar = 100 µm).

Note: Tw-20 was eliminated from additional staining groups because it required more time than usual, did not generate impressive outcomes in H&E, and had fragmented ECM and widespread cytoplasm. However, it was examined for the quantification of collagen and sGAG to check whether there was any distinction between its ID and PD modes. On the other hand, CHAPS also took a long time to decellularize, but its ECM survived unchanged; therefore, it was subjected to additional testing for staining and quantification of sGAG and collagen.

The nuclear content was estimated by quantification and DAPI staining, but residual cytoplasm could not be estimated by that; hence H&E staining was done to check whether cytoplasm had been left behind by these detergents in both ID and PD set-ups. Besides DNA, residual cytoplasm might also be detrimental to the final scaffold. Residual cytoplasm can impair the ability of cells to repopulate the scaffold and develop new tissue, weaken the scaffold, make it less resistant to mechanical stress, and alter the composition of the extracellular matrix (ECM), thus further distorting the scaffold’s mechanical and biochemical properties. Any cellular residues, such as DNA, mitochondria, cytoplasm membrane, lipids, and cytosolic material, may have an inflammatory effect on the recipient if not thoroughly eliminated (Naba et al., 2015). Upon H&E staining, the native tissue revealed all 3 cell types characteristic of the pancreas, namely, islets-stained faint pink (Figure 4A), closely packed acinar cells-stained dark pink (Figures 4B), and nucleus–stained purple (Figure 4B). In contrast, the DT should lack all three types of cells and possess only the mesh-like structure of the clear ECM. This ECM structure was obtained only with TX-100 in PD (Figure 4H). SDC and SDS have also achieved this structure in PD (Figures 4J, L); however, the ECM perimeter is more noticeable in TX-100 DT. TX-100 predominantly attacks lipid-lipid and lipid-protein interactions, keeping protein-protein interactions unaltered; hence it has shown a clear ECM network after decellularization (Moffat et al., 2022). In contrast, a significant amount of cytoplasm was noticed to be scattered across the ECM, especially in Tw-20 (Figures 4C, D) and CHAPS tissue (Figures 4E, F), both in ID and PD. Since Tw-20 is a mild detergent known to solubilize lipids and proteins under specified conditions, it was recognized that it decellularizes tissue slowly and leaves cytoplasm behind, making it an unsuitable option for decellularization (Chaschin et al., 2022). In ID, TX-100 exhibited shrunken cytoplasm, which could be seen adhering to the broken ECM network (Figure 4G), while SDS (Figure 4I) and SDC (Figure 4K) have the same shrunken cytoplasm accompanied with discrete strands of ECM. These ECM strands were found to be broken in SDC (Figure 4K) while it was intact in SDS (Figure 4I). Among all these, the best-performing detergent was TX-100 in PD which had a distinct ECM perimeter (Figure 4H) with no remnant DNA or cellular content. This meticulous ECM perimeter was also achieved with SDS (Figure 4J), although the boundaries of the ECM were somewhat obscure. These boundaries were marginally improved in SDC (Figure 4L). The requisite ECM profile was attained in PD with TX-100, SDS, and SDC. Similar to SDS, SDC eliminates cellular material by dissolving the cell membrane, releasing the cellular content all at once with no immunogenic residues (Alshaikh et al., 2020). A similar trend was observed with the pig urinary bladder matrix, where SDC and TX-100 produced scaffolds with an intricate fibrous ECM network, whereas CHAPS negatively altered the ECM (White et al., 2017).

### 3.4 sGAG imaging

**FIGURE 5:**
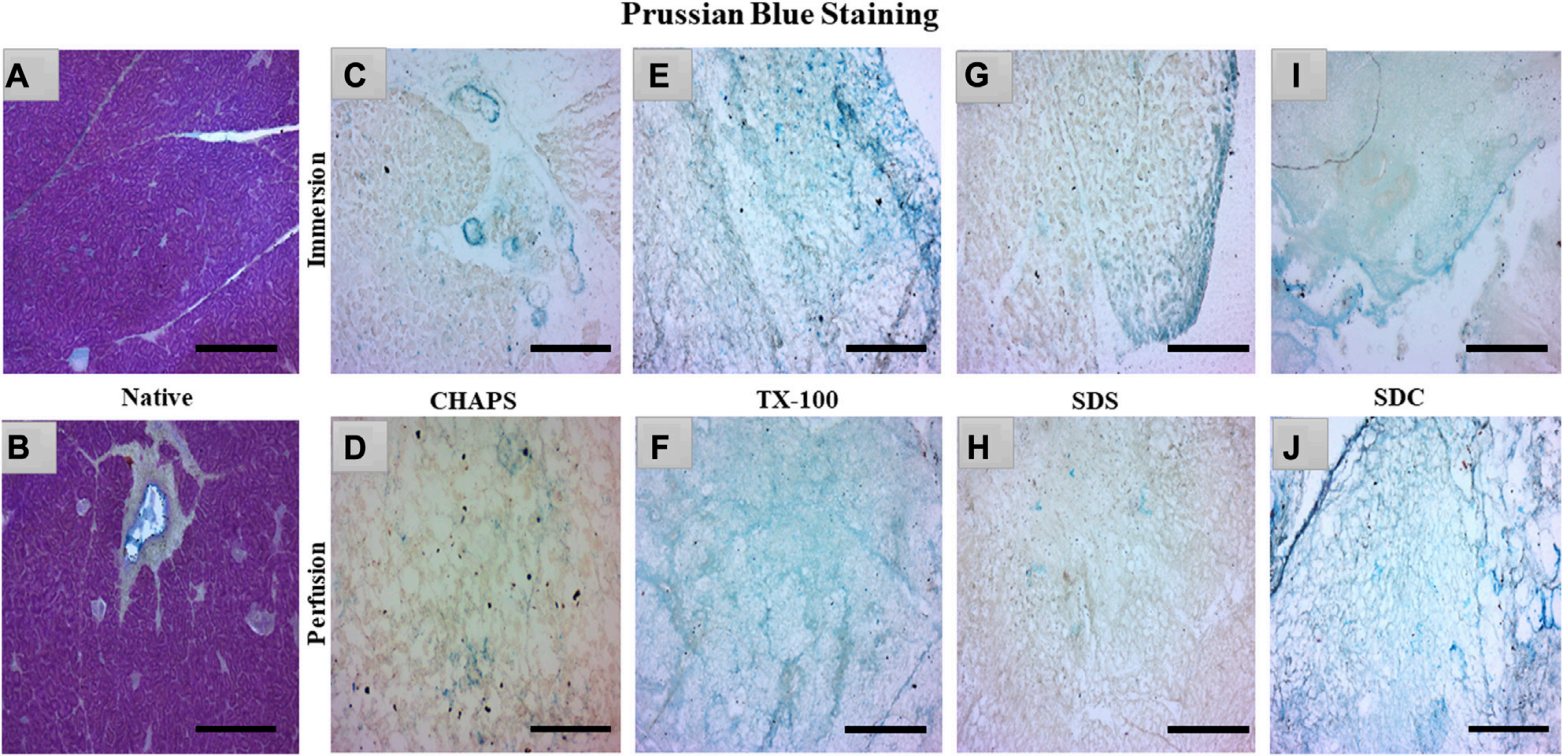
In the native tissue, sGAG content (a, blue) was seen across the associated ECM network **(A)** and surrounding the pancreatic ducts **(B)**. As for CHAPS, the sGAG content was detected to be sporadically present in ID (**(C)**-blue stained blots) and minimum staining was obtained in PD (**(D)**-faint blue staining along the perimeter of ECM). TX-100 has demonstrated widely spread sGAG content in **(E)** ID, which was found to be more prominent in PD (**(F)**, blue stained ECM perimeter). In ID, SDS exhibited lesser staining (**(G)**, blue stained tissue at the edges) than TX-100, whereas, in PD, the difference was negligible (**(H)**, tissue’s ECM stained brown without any trace of blue stain). SDC has preserved the maximum sGAG content in ID accompanied by some smeared cytoplasm (**(I)**, blue stain on the tissue border lines with brown stained cytoplasm) additionally, in PD, the sGAG content was clear, and the ECM structure was visible (**(J)**, dark blue stained tissue edge while the ECM stained blue at the intermittent presence of sGAGs). (Scale bar = 100 µm).

sGAG is crucial to examine after decellularization since it provides directional cues and strong adhesion sites for recellularized cells as they interact with various growth factors, cytokines, and cell surface receptors, influencing cellular proliferation for tissue regeneration (Ergun et al., 2022). Due to their sulfate groups, sGAGs have a strong negative charge, which can attract and bind water molecules, leading to their water retention properties (Herrera Quijano et al., 2022). This can assist in maintaining an optimal level of hydration in the ECM and scaffold, which is vital for cell longevity and functionality. GAG are extraordinarily hydrophilic and, as a result, adopt highly stretched conformations that enable matrices to withstand high tensile stress (Frantz et al., 2010). To verify the existence of sGAG following decellularization, the remaining sGAG content was stained blue using Prussian blue. In the native tissue, the intercellular gaps were stained blue, indicating the presence of sGAG (Figure 5A); this stain became darker around the pancreatic ducts, suggesting a higher proportion of sGAG (Figure 5B). Subsequently, in CHAPS, the presence of remnant sGAG was relatively scanty in ID and could be detected as brown spots (Figure 5C), and a similar pattern was observed in its PD set-up (Figure 5D). TX-100 has shown an abundance of sGAG in both ID and PD (Figures 5E, F). CHAPS is weak and non-disordering in its interaction with lipid membranes, whereas TX-100 has been described as intense and disordering, thus leading to better retention of sGAG with TX-100 (Rodi et al., 2014). SDS has a cytoplasmic presence as brown smears and has shown sGAG only on the edges in ID (Figure 5G) and almost none in PD, while ECM of PD was intricately built (Figure 5H). The fundamental explanation for this is that sGAG in the ECM of the caprine pancreas cannot tolerate the SDS concentration we applied and was easily washed away. Similar to SDS, SDC has demonstrated the presence of sGAG around the margins of tissue in ID (Figure 5I), but unlike SDS, SDC has demonstrated the abundant presence of sGAG throughout the ECM in PD with unaltered ECM architecture (Figure 5J). Through sGAG imaging, it is reasonable to infer that only two detergents, i.e., TX-100 (in both ID and PD) and SDC (in PD), could fulfill the required scaffold parameter of sGAG.

### 3.5 sGAG Content estimation

**FIGURE 6:**
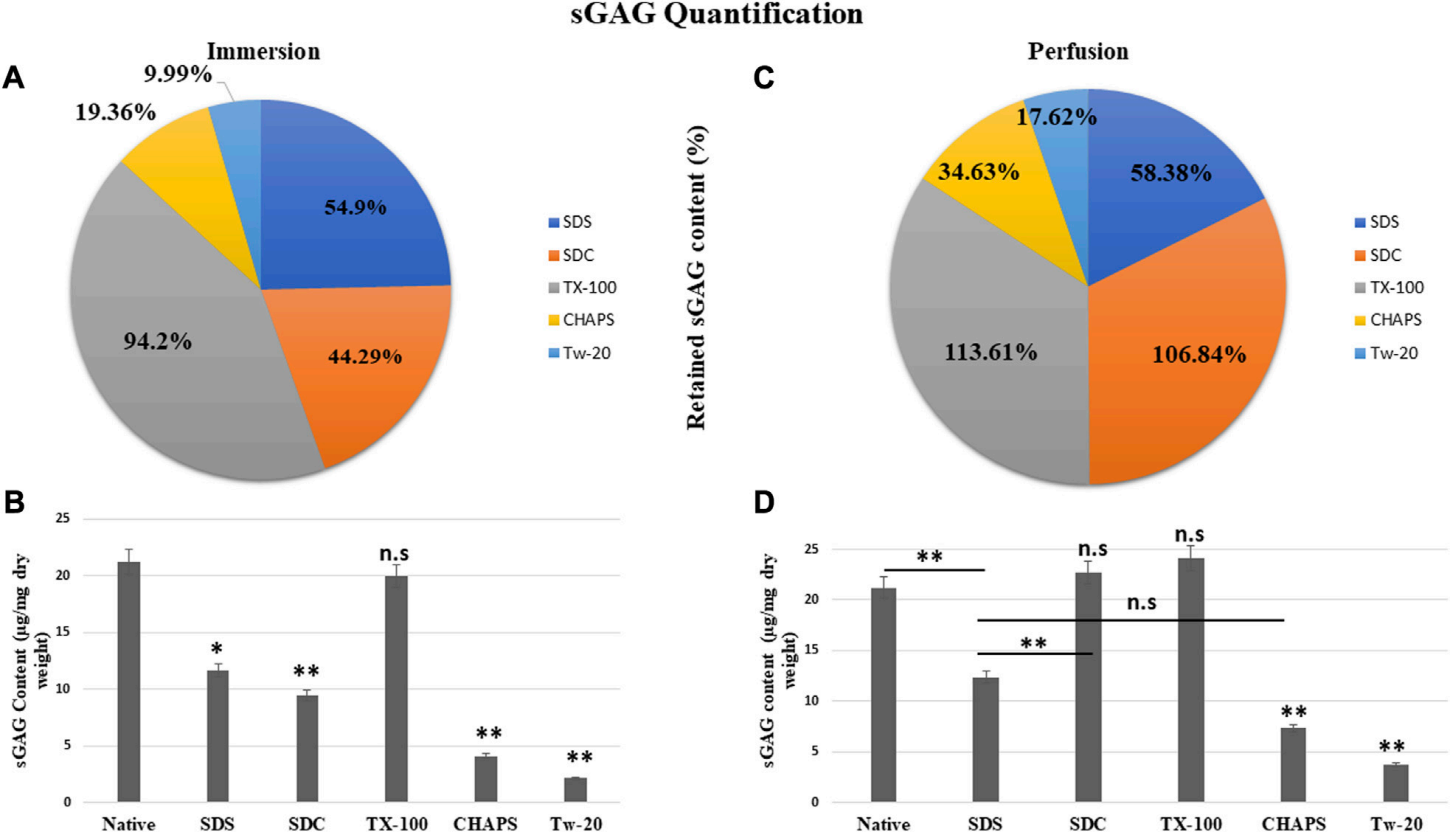
The quantity of retained sGAG by all the detergents was evidently greater in PD **(B)** than in ID **(A)**. SDC from PD (b, orange) had shown a positive difference with 62.55% more retained sGAG content than SDC from ID (a, orange), while other detergents performed marginally better in PD **(C)**. Quantification of the sGAG content in ID **(B)** revealed that only TX-100 displayed no significant difference (*p* > 0.05) from the native, unlike SDS (*p* < 0.05), SDC, CHAPS, and Tw-20 (all three with *p* < 0.001) that exhibited considerable variations. In PD, the quantification of sGAG suggested that both TX-100 and SDC **(D)** retained sGAG satisfactorily (*p* > 0.05), while SDS, CHAPS, and Tw-20 (all three with *p* < 0.001) continue to lag behind in preserving the sGAG. Among the ineffective detergents in PD **(D)**, SDS and CHAPS performed comparably (*p* > 0.05) in preserving sGAG while Tw-20 (*p* < 0.001) was the least effective among all.

The residual content of PD detergents was reported to be significantly higher than that of ID detergents (Figures 6A, C). As observed from quantification with DMMB assay, only TX-100 was able to preserve the sGAG 19.99 ± 0.92 μg/mg of dry tissue weight in ID (Figure 6B), showing no significant difference (*p* = 0.964) than native, which had 21.22 ± 1.89 μg/mg. All other detergents experienced a decline in this parameter during ID. The sGAG amount in SDS = 11.65 ± 0.638 μg/mg was close to SDC, which had 9.4 ± 1.3 μg/mg of sGAG (*p* = 0.701), while the SDS amount (Figure 6B) ended up going drastically different from TX-100 (19.99 ± 0.92 μg/mg, *p* = 0.002), CHAPS (4.11 ± 0.69 μg/mg, *p* = 0.004), and Tw-20 (2.12 ± 0.66 μg/mg, *p* = 0.001). When perfused through tissue, the same detergents’ concentration exhibits excellent sGAG retention characteristics (Figure 6B), establishing conclusively that not only the detergent concentration but also its mode and exposure duration influence the integrity of the final decellularized ECM. The detergents even exposed the sGAG concealed between the cells and ECM, resulting in more than 100% retention of sGAG in the case of SDC and TX-100 (Figure 6B). TX-100 ensured the presence of ample sGAG in both ID and PD. None of the other three detergents in PD, namely, SDS, CHAPS, and Tw-20, exhibited the same improved sGAG retention percentage. Among these lagging three detergents in PD, CHAPS has an improved sGAG retention, while the other two detergents performed ordinarily compared to their ID counterparts. In PD, detergents did better at retaining the sGAG, with only SDS, CHAPS, and Tw-20 lagging behind (Figure 6D). H&E staining implies that the deteriorated ECM in these PD detergents could cause this behavior; as the ECM disintegrated, it dragged away the sGAG bound onto its surface. SDC and TX-100 maintained nearly the same levels of sGAG as the native in PD, which was 22.67 ± 1.3 μg/mg, 24.11 ± 0.68 μg/mg, and 21.22 ± 1.89 μg/mg resp. SDS, CHAPS, and Tw-20 had retained only 12.39 ± 0.62 μg/mg, 7.35 ± 0.64 μg/mg, and 3.74 ± 0.71 resp, while SDC and TX-100 have shown no significant reduction in sGAG amount, as they had *p* = 0.928 and *p* = 0.463 resp from the native. On the other hand, SDS (*p* = 0.001), CHAPS (*p* = 0.00001), and Tw-20 (0.0000)1 have shown highly significant reductions in sGAG amount. In contrast to other detergents, the ID and PD performance of TX-100 was held steady.

### 3.6 IHC staining

**FIGURE 7:**
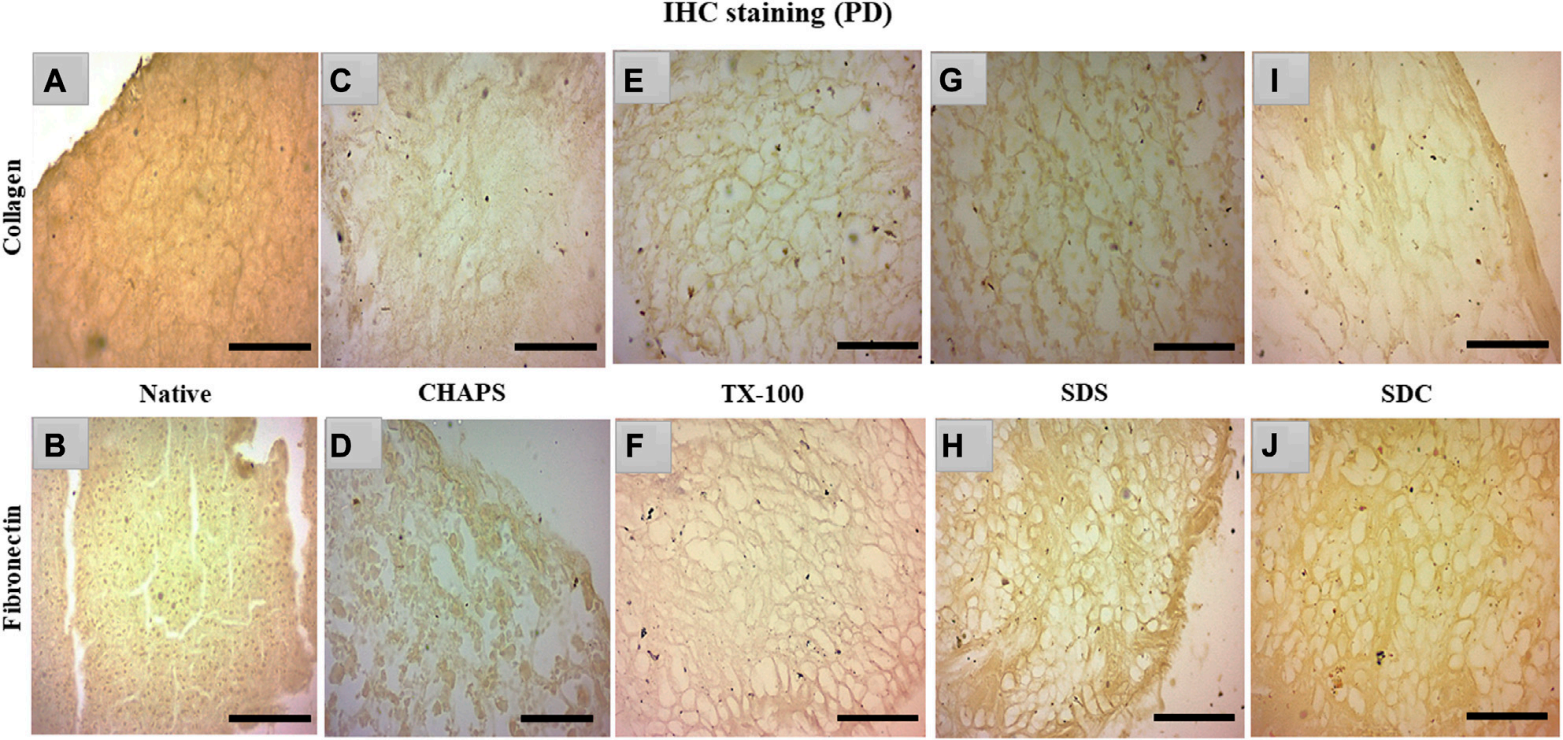
IHC staining of the native tissue indicated the existence of collagen **(A)** between the cell margins and fibronectin **(B)** at the tissue edges. The CHAPS detected collagen in the disseminated ECM **(C)** and fibronectin at the tissue margins as well as in the scattered cytoplasm **(D)**. TX-100 has retained evenly distributed collagen **(E)**, however its fibronectin level is not as abundant (**(F)**, stained slightly brown) as its collagen content. SDS indicated fragmented collagen fibrils **(G)** and dense fibronectin at the tissue boundaries **(H)**. SDC has preserved thick collagen fibrils at the borders **(I)**, and fibronectin was observed as uniformly dispersed **(J)**. (Scale bar = 100 µm).

Collagen and fibronectin are the most vital pancreatic matrix proteins crucial for tissue architecture and facilitate cell proliferation (Naba et al., 2017). Fibronectin is a prevalent ECM glycoprotein aggregated into a fibrillar matrix across all tissues throughout all life stages (Singh et al., 2010). Most of the ID tissues lacked intact ECM or had faded ECM, rendering the stain undetectable even with the imaging software’s lowest brightness and maximum contrast. In addition, the ID retained the cytosolic content, an undesirable trait in a scaffold; as a result, these samples were excluded from immunohistochemical staining. Collagen and fibronectin staining has only been observed in PD. Collagen isoforms in the ECM were barely detectable in the native tissue because it was concealed underneath the native cells, but a dark brown interconnected network was observable (Figure 7A). Similarly, fibronectin was exclusively expressed near the borders of native tissue (Figure 7B). In CHAPS tissue, the collagen was found in diffused form across the tissue with no discernible ECM network perimeter (Figure 7C). Similarly, fibronectin was observed to be weakly stained at the tissue margins in this case (Figure 7D), along with smeared cytoplasm. TX-100 had a remarkable amount of collagen content with interspersed ECM network (Figure 7E) but far less fibronectin, as this readily took the stain (Figure 7F). If fibronectin is expected as the final outcome in the caprine pancreatic scaffold, then TX-100 is not an effective detergent. While SDS had collagen, it was seen as being smeared along with a faint cytoplasmic hint (Figure 7G), while fibronectin was seen present at the edges of this tissue with an intact ECM network (Figure 7H). Low concentrations of SDS are known to successfully safeguard collagen in earlier decellularized scaffolds (Xing et al., 2015). This detergent’s high concentration forms scaffolds with poor collagen concentration; hence, its concentration and exposure period limit its applications. Collagen was visible on the borders of SDC (Figure 7I), whereas fibronectin was ascertained across the ECM (Figure 7J). SDC is a gentle detergent that is reported not to impair the ECM of tissues during decellularization (Philips et al., 2022); hence it has successfully retained both major ECM proteins, i.e., collagen (on the tissue edges) and fibronectin. On the contrary, Milan et al. reported that SDC alone showed no success in retaining collagen, but in our study, it has retained the collagen on tissue edges (Milian et al., 2021). Besides this study, numerous other xenogeneic tissues have also exhibited superior results with TX-100, such as porcine aortic valves (GRAUSS et al., 2005), porcine pancreas (Klak et al., 2021; Zhu et al., 2021), and porcine liver (Mirmalek-Sani et al., 2013), etc. Whereas in other investigations, SDS has been favored over TX-100 for decellularization of densely fibrous tissues such as kidney and liver, as TX-100 has demonstrated poor outcomes (Hussein et al., 2016).

### 3.7 Collagen quantification

**FIGURE 8:**
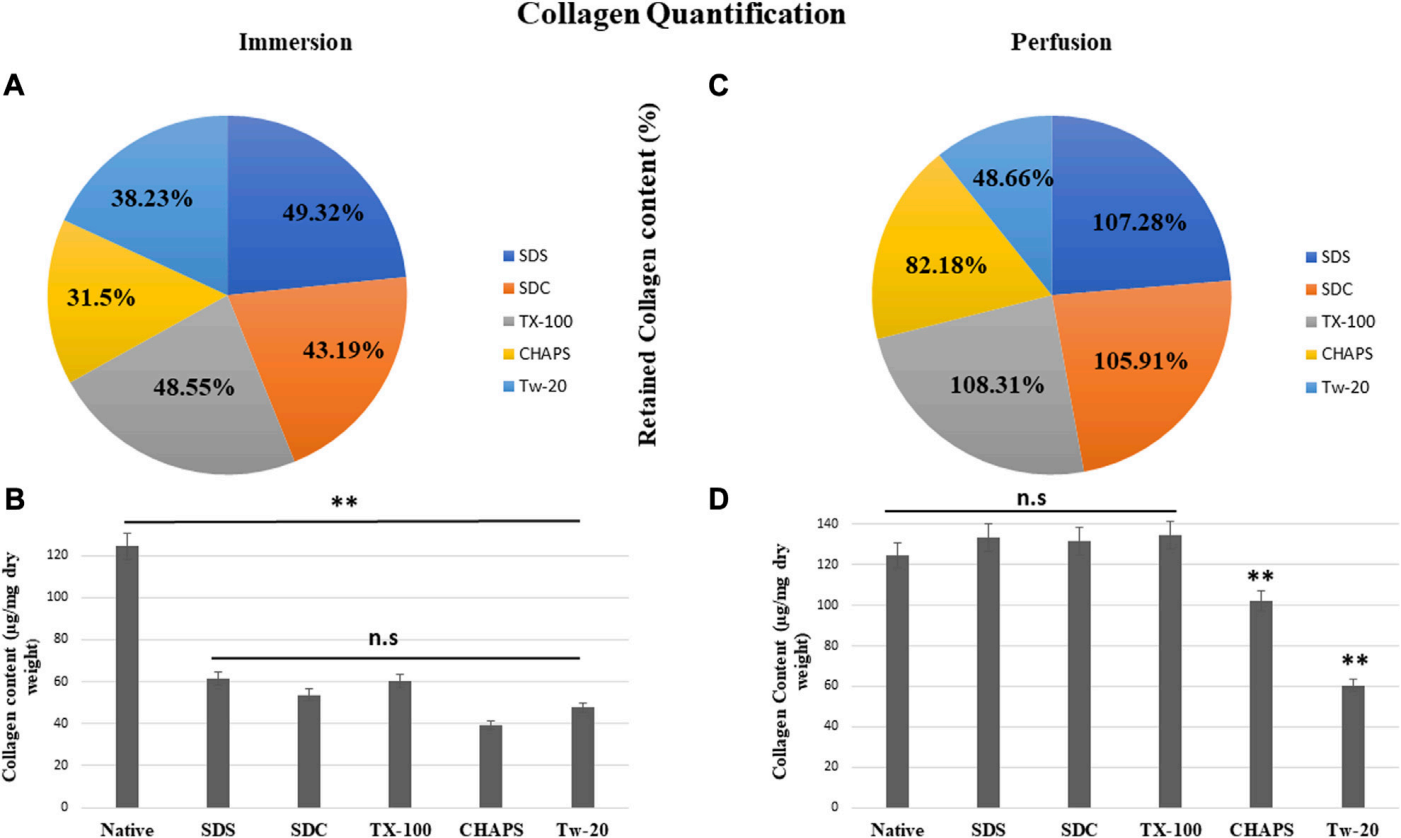
All ID detergents collectively demonstrated poor collagen retention **(A)**, whereas their PD competitors indicated substantially enhanced retention **(C)**. Except for Tw-20, which showed just a 10.23% boost in PD **(C)**, all other detergents in PD enhanced the proportion of collagen retention by 50%–60%. SDC was the top of this retention with 62.72% improved retention in PD **(C)**. Upon collagen quantification, ID-treated tissues showed **(B)** no statistical differences (*p* > 0.05) among themselves, but a statistically significant difference was noted (*p* < 0.001) from native tissue. In the case of PD, collagen retention was remarkably adequate (*p* > 0.05) in SDS, SDC, and TX-100 **(D)**, however CHAPS and Tw-20 were unable to retain collagen (*p* < 0.001) even under the PD setting.

According to reports, among all the matrisome proteins, collagen subunits govern numerous cellular functions directly via integrin-mediated signaling (Berger et al., 2020). Collagen is the most critical matrix protein to quantify, as it is responsible for the mechanical attributes, organization, and shape of decellularized tissues. After reseeding the scaffold, collagen plays a crucial role as this protein interacts with cells via multiple receptor families and regulates their multiplication, migration, and maturation (Ricard-Blum, 2011). As observed with sGAG retention, collagen quantification demonstrated consistent results, as all detergents retained collagen better in PD. In ID, CHAPS and Tw-20 trailed behind, while the collagen retention of the other three detergents was superior (Figure 8A). Quantified collagen in native tissue was found to be 124.31 ± 7.21 μg/mg dry tissue weight, while the collagen estimated in other detergents of ID was SDS = 61.32 ± 4.64 μg/mg, SDC = 53.7 ± 8.01 μg/mg, TX-100 = 60.36 ± 3.28 μg/mg, CHAPS = 39.16 ± 2.94 μg/mg, Tw-20 = 47.53 ± 12.2 μg/mg (Figure 8B). All of the detergents in ID omitted 50% of the collagen content, which is an enormous loss and a significant deviation from the original tissue (*p* < 0.00001). Conversely, PD has revealed that the same detergent concentration retains more than 100% of collagen deposition. In ID, extended exposure to the detergents caused ECM to become fragmented and smeared, resulting in the significant mass of collagen washing away, whereas, in PD, the exposure time was shortened, allowing the detergents to immediately eliminate the cells and preventing them from interacting with ECM for a longer duration. Except for Tw-20 (60.49 ± 0.62 μg/mg; *p* = 0.0001) and CHAPS (102.16 ± 1.18 μg/mg; *p* = 0.008) (Figure 8D), all detergents performed better in PD with no significant difference in collagen content than native (SDS = 133.36 ± 2.7 μg/mg; *p* = 0.485, SDC = 131.66 ± 2.04 μg/mg; *p* = 0.680, TX-100 = 134.65 ± 2.87 μg/mg; *p* = 0.354), CHAPS = as all these retained more than 50% of collagen. Tw-20 merely upheld 10% more collagen than its ID counterpart (Figure 8C). This increased proportion of collagen following decellularization can be ascribed to the revelation of previously concealed ECM structure revealed after cell removal, raising the overall collagen content in the quantitative analysis. Excessive sterilization procedure is also known to diminish these components, and we did not undertake rigorous sterilization procedure, which explains the surplus amount of Collagen and sGAG achieved after decellularization (Klak et al., 2021). Another study involving the decellularization of bovine cartilage yielded similar results showing higher collagen content after decellularization with SDS (Utomo and Sari, 2019). Similarly, the collagen was found to be six-fold higher in the DT than the native tissue in porcine optic nerve after decellularization with TX-100 (Sun et al., 2020). Hence, it is quite common to obtain a higher ECM protein content than in native, and the underlying cause might be that these proteins become unmasked after decellularization.

### 3.8 SEM imaging

**FIGURE 9:**
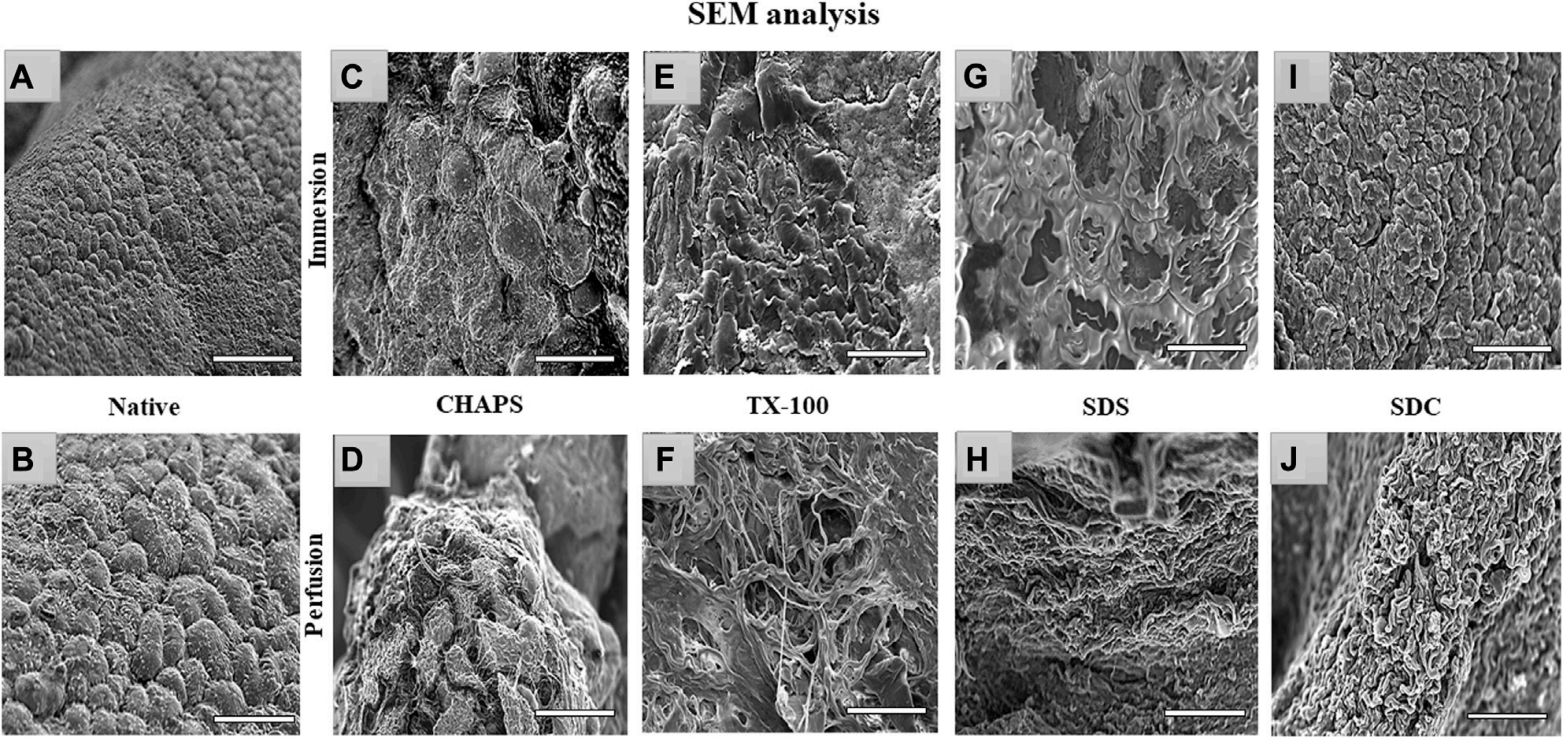
Under SEM, the native tissue was seen with cells protruding out of the surface **(A, B)**; similar bulging-out cells were also observable with CHAPS **(C, D)**, indicating inadequate decellularization. In case of TX-100, fibrillar collagen structure was recognized **(F)** in PD, but a similar result was not obtained in its ID equivalent, which had an indication of leftover cells’ presence that were protruding out **(E)**. ID of SDS had distinct boundaries **(G)**, but a cellular presence can be detected beneath the interconnected ECM fibers, while in the PD counterpart of the same had displayed fibrillar form of intertwined collagen **(H)**. With SDC ID, the native cells were observed to be projecting out **(I)** but their PD counterpart had a distinct collagen fibril mass **(J)**. (all scale bar = 100 μm, and scale bar for a) = 50 µm).

Through SEM imaging, it was verified if the fibrillar architecture of the ECM network in the DT was intact. The presence of native cells with a structure like a cluster of grapes, which is typical of pancreatic cells (Chen et al., 2004), has been demonstrated in native tissue (Figures 9A, B). However, in ID, the majority of these detergents failed to completely eliminate the cells, as little protruding structures were observed in each of them (Figures 9C, E, G, I). These minor protruding patterns in ID have validated what was estimated from H&E staining (Figures 4E, G, I, K) of these tissues. Out of all the ID SEM micrographs, only SDS (Figure 9G) exhibited distinctible ECM outlines, but these also indicate the residual cytosolic content concealed between these ECM perimeters, as detected with H&E staining as well (Figure 4I). The emergence of these types of structures in SEM images of ID has confirmed what was estimated from H&E staining of similarly treated tissues. In PD, only CHAPS (Figure 9D) exhibited this bulging surface, whereas TX-100, SDS, and SDC presented a distinct interconnected fibrillar ECM microstructure, which is a prerequisite for a satisfactorily decellularized scaffold (Figures 9F, H, J).

### 3.9 Swelling index study

**FIGURE 10:**
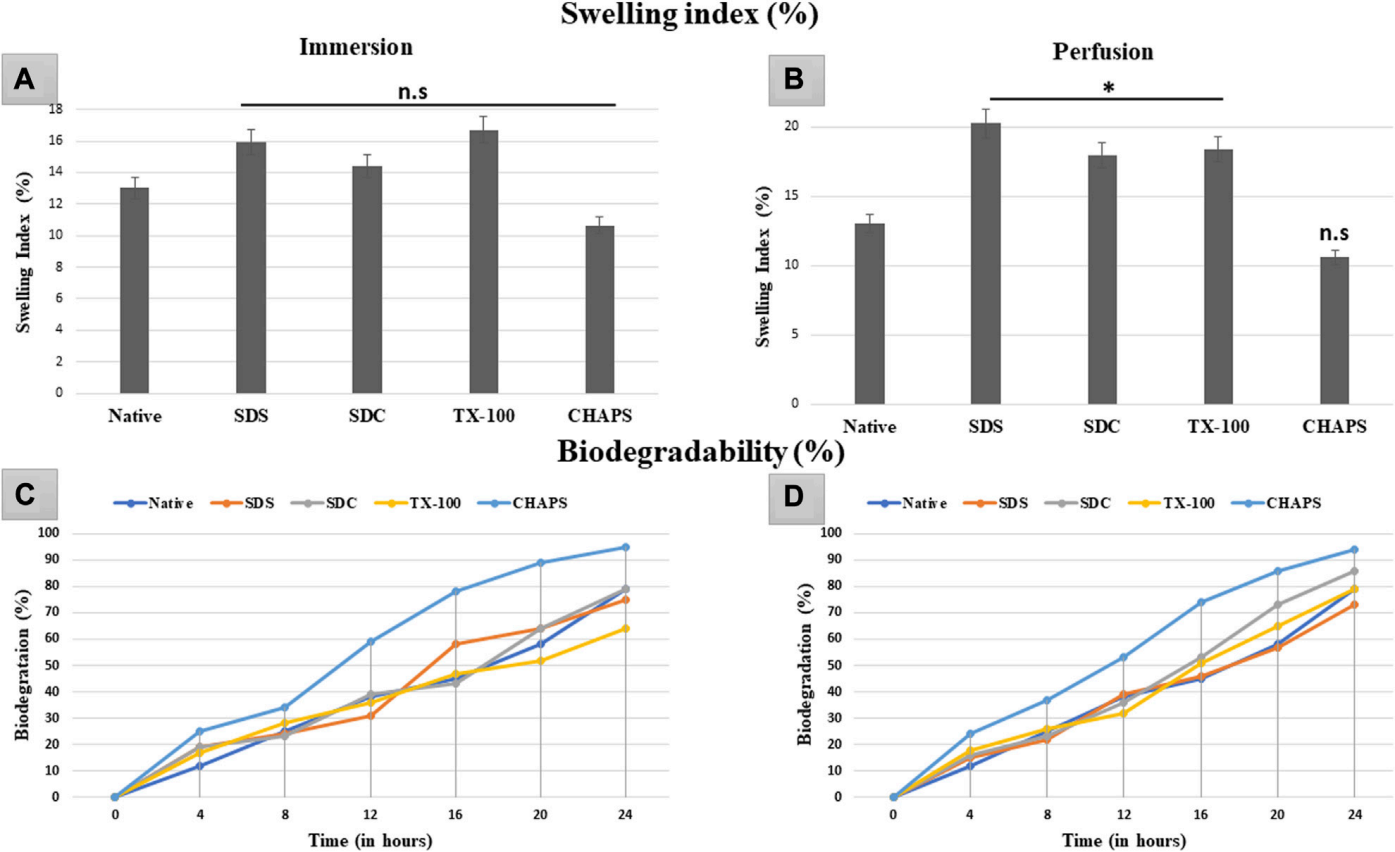
The swelling index study revealed that ID-tissues have **(A)** retained water content almost identical (*p* > 0.05) to that of native tissue. Similarly, PD-tissues **(C)** absorbed higher water percent (*p* < 0.05), except for CHAPS which absorbed water almost equivalent to the native tissue (*p* > 0.05). Considering the biodegradability in ID, CHAPS tissue deteriorated at a significantly faster pace and in less time, while it is PD counterpart exhibit the similar result. Other detergent-treated tissues in ID have decomposed comparably to native tissue **(B)**, with the exception of SDS, which degraded 60% within 16 h. For PD, the biodegradation outcomes were almost similar, except that **(D)** SDS tissue degraded at the same pace as native tissue, unlike in ID.

The swelling index indicates the scaffold’s porosity or the degree of interconnected void spaces (Loh and Choong, 2013). A scaffold with improved porosity facilitates the flow of nutrients, oxygen, and waste products, which are essential for cell survival and tissue regeneration (Bhatt et al., 2022). Therefore, calculating the swelling index can help determine the scaffold’s porosity and its potential for supporting cell growth and tissue regeneration. The DT should be capable of preserving high structural strength under physiological conditions without extensive swelling or shrinkage. In ID, the swelling index obtained with the detergents SDS (15.92% ± 0.09%, *p* = 0.35), SDC (14.39% ± 0.59%, *p* = 0.91), TX-100 (16.74% ± 1.54%, *p* = 0.15) and CHAPS (10.63% ± 0.49%, *p* = 0.55) demonstrated statistically insignificant difference from the native tissue (13.01% ± 1.22%) (Figure 10A). While in PD, SDS (19.44% ± 0.98%, *p* = 0.004), SDC (16.95% ± 2%, *p* = 0.042), and TX-100 (18.37% ± 0.73%, *p* = 0.027) has absorbed significantly high volume of water, whereas CHAPS (10.09% ± 0.28%, *p* = 0.49) sustained its results like ID (Figure 10B). The swelling index of detergents in ID is comparable to that of native detergents, whereas detergents in PD settings depict a significantly greater swelling index. A plausible justification behind this observation is that the native tissue lacked the voids, similar to DT, to absorb and retain water. In contrast, ID tissues had fragmented ECM (as evident from H&E staining) as well as residual cells (as seen in its SEM tissues), which limited the water uptake, hence displaying a similar swelling index to that of native tissue. On the other hand, PD tissues have perfectly intact ECM networks with voids that upheld higher water absorption, which could not flow out easily due to intricate ECM architecture. As stated previously, the higher the porosity, the higher the swelling index, which accelerates the exchange of nutrients; hence it is a favorable alteration in the scaffold. Overall, the caprine pancreatic tissue has shown better swelling index in PD than in an ID set-up.

### 3.10 Biodegradability analysis

In tissue engineering, the biodegradation rate of DT can be a crucial parameter to consider in tissue engineering, as it can impact the timeline of tissue regeneration and replacement (Berteanu et al., 2016). If the scaffold deteriorates too rapidly, it may not provide adequate structural integrity and microenvironmental cues required for cell adhesion, growth, and function. Conversely, if the scaffold degrades too slowly, it may impede the regeneration of new tissue or induce a long-term foreign body response (Hussein et al., 2017). The rate of biodegradation of ID was equivalent to that of native tissue, except for CHAPS (Figure 10C), which deteriorated excessively (60%) in a much shorter time (12 h). After 24 h, only TX-100 (64%) and SDC (79%) showed a parallel disintegration rate with that of native (80%), while SDS started degrading at a markedly faster pace after 12 h. CHAPS made significant progress in PD (53% in 12 h) than in ID (60% in 12 h), although its biodegradation rate was still higher than that of competing detergents; hence this detergent is unsuitable for decellularizing the caprine pancreatic ECM. Apart from CHAPS, maximum degradation in PD was found with SDC, which degraded 86% after 24 h, as compared to SDS (73%) and TX-100 (79%) during the same time span. The anticipated output for biodegradation has been accomplished by PD detergents, namely, SDS and TX-100.

### 3.11 Tensile property test

**FIGURE 11:**
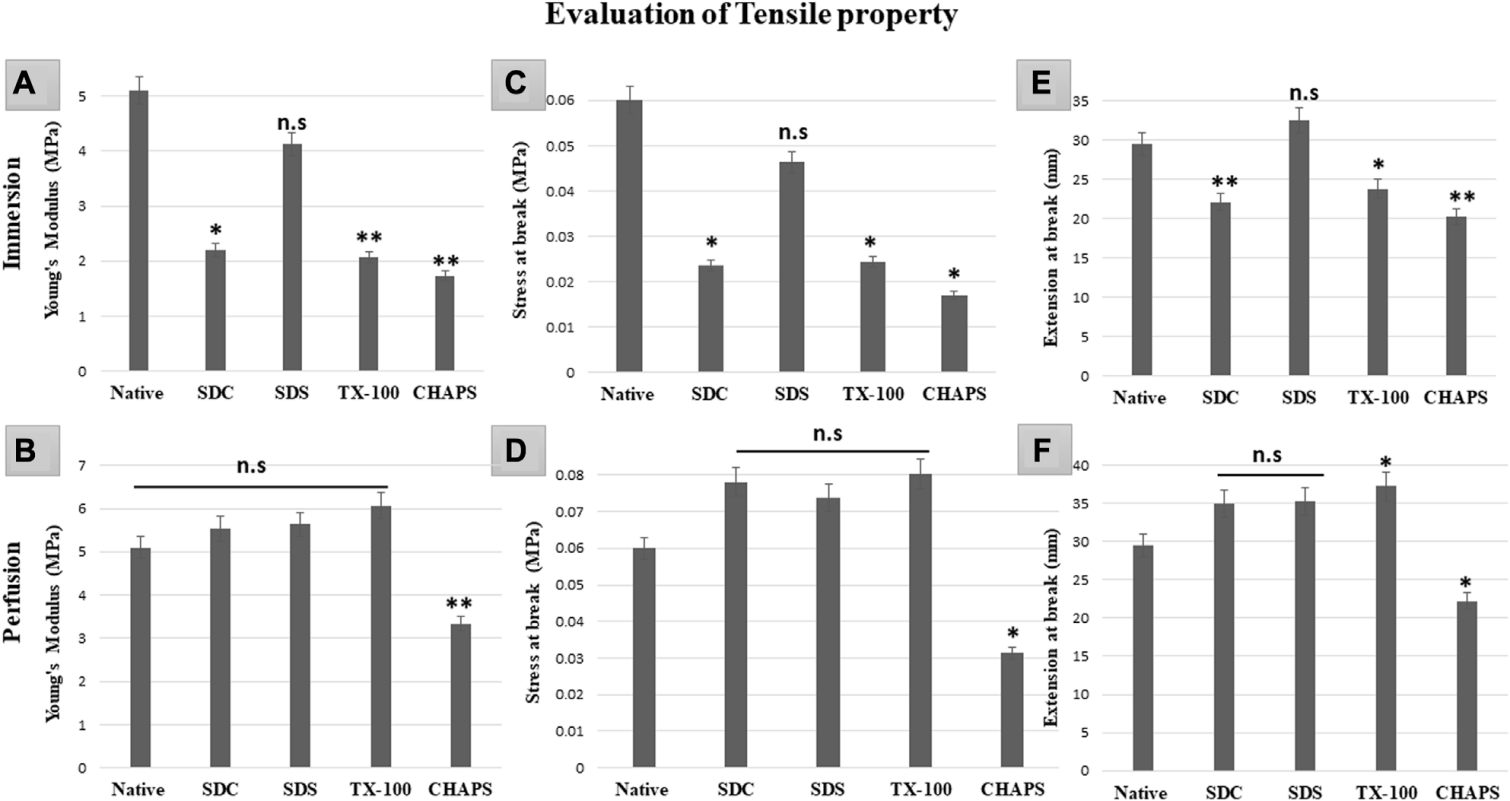
It was determined that the overall tensile property of PD-tissues **(B,D,F)** was superior to that of ID-tissues **(A,C,E)**. Y. M of SDS in ID **(A)** was the only one to be at par with that of native tissue (*p* > 0.05), while SDC and TX-100 coupled with CHAPS shown highly significant differences (*p* < 0.001). While Y. M in PD showed only CHAPS to be significantly different (*p* < 0.001) from the native, the remaining detergents have shown no significant decline (*p* > 0.05) in Y.M. The stress at break in ID **(C)** indicated that SDS performed significantly better as it was at par with the native tissue (*p* > 0.05), as compared to SDC, TX-100, and CHAPS (all three had *p* < 0.05) for this attribute. Only CHAPS failed (*p* < 0.05) to maintain the stress at break parameter in PD **(D)**, whereas SDC, SDS, and TX-100 were able to preserve the stress at break parameter (*p* > 0.05) of the native tissue. The extension at break in ID **(E)** was found to be statistically equivalent to the native tissue in SDS (*p* > 0.05), but TX-100 (*p* < 0.05), SDC, and CHAPS (*p* < 0.001 for both) showed substantial deviations. In contrast to ID, the extension at break in PD **(F)** has demonstrated superior efficacy with SDC and SDS, whereas TX-100 has shown excessive elongation and CHAPS had considerably lesser elongation than native (*p* > 0.05 for both).

Mechanical tests of DT establish whether the scaffold has high durability and structural stability to withstand the physiological stress it may encounter *in-vivo*, post-implantation (Neishabouri et al., 2022). If the scaffold collapses under stress, it may not be suitable for the intended tissue engineering application as it may not be able to maintain its internal microstructures throughout tissue regeneration or function (Kanda et al., 2023). Therefore, it is of the utmost priority that the scaffold’s mechanical properties strongly resemble that of the original tissue since this would be essential for supporting optimal tissue function and limiting any detrimental impact, such as mechanical mismatch or tissue injury. In ID, all the detergents failed to retain the elasticity as shown by the significantly lower Young’s modulus (Y.M) of each tissue compared to the native (5.1 ± 0.208 MPa) except for SDS (4.1 ± 0.49MPa, *p* = 0.63). In comparison, SDC (2.2 ± 0.55 MPa, *p* = 0.012), TX-100 (2.06 ± 0.35MPa, *p* = 0.009), CHAPS (1.73 ± 0.66MPa, *p* = 0.004) have shown statistically significant variations from native (Figure 11A). Nevertheless, marginally better findings were achieved on estimating the Y. M for PD detergents (Figure 11B) excluding CHAPS (3.33 ± 0.42 MPa,*p* = 0.006), which was still lagging in the Y. M of DT. In P. D, no statistically significant difference was recorded in Y. M of SDS (5.6 ± 0.26 MPa, *p* = 0.639), SDC (5.53 ± 0.14 MPa, *p* = 0.783) and TX-100 (6.06 ± 0.21 MPa, *p* = 0.156) when compared to native (5.1 ± 0.208 MPa). The stress at break, also known as the ultimate tensile strength, of a DT represents the maximum amount of stress or force the scaffold can endure before it splits into two pieces (Yavari Maroufi and Ghorbani, 2022). It measures a structure’s resistance to deformation and breakdown when exposed to a tensile load. The stress at break indicates the scaffold’s ability to maintain its structural integrity and resist failure under applied tensile loads. Higher stress at break value implies a stronger material that is less prone to rupture severely, which might be beneficial for tissue engineering applications that require mechanical stability and durability (An et al., 2018). In ID, with the exception of SDS (0.046 ± 0.009MPa, *p* = 0.68) (Figure 10C), it was discovered that the stress at break for all the detergents, namely, SDC (0.02 ± 0.005 MPa, *p* = 0.034), TX-100 (0.024 ± 0.005 MPa, *p* = 0.038), and CHAPS (0.017 ± 0.003 MPa, *p* = 0.013) varied greatly from the native (0.06 ± 0.009 MPa). Whereas in PD SDC (0.07 ± 0.005 MPa, *p* = 0.311), SDS (0.07 ± 0.002 MPa, *p* = 0.553), TX-100 (0.08 ± 0.007 MPa, *p* = 0.217) have shown better stress at break in PD (Figure 11D), except for CHAPS (0.03 ± 0.001 MPa, *p* = 0.04). The extension at break displays the scaffold’s ductility, which is its capacity to undergo plastic deformation or elongation without fracture (Püllen et al., 2021). A higher extension at break value indicates that the scaffold can stretch or deform extensively before fracturing, which can be important for tissue engineering applications that require flexibility or compliance (Reing et al., 2010). The maximum extension at break was shown by SDS (32.55 ± 2.12 mm, *p* = 0.422) in ID (Figure 11E), which was statistically insignificant from native tissue (29.53 ± 0.96 mm), while other detergents like SDC (22.11 ± 0.7 mm, *p* = 0.009), TX-100 (23.7 ± 1.01 mm, *p* = 0.039) and CHAPS (20.29 ± 0.14 mm, *p* = 0.002) exhibited far less extension. Hence, the ID set-up was not found to be suitable for overall mechanical strength of the caprine pancreatic ECM scaffold. Although in PD, this extension at break expanded marginally for all detergents except CHAPS, which produced a reduced extension at break, whilst TX-100 demonstrated an unusually enhanced extension at break. In PD, SDC (34.95 ± 1.95 mm, *p* = 0.113) and SDS (35.28 ± 1.54 mm, *p* = 0.087) had moderately increased extension at break (Figure 11F) with no significant difference, while TX-100 (37.25 ± 1.03 mm, *p* = 0.018) and CHAPS (22.19 ± 1.18 mm, *p* = 0.025) had a highly significant difference in the length of extension at break from that of native (29.53 ± 0.96 mm). This finding reveals that if PD setup is used, then SDC, SDC, and TX-100 will perform remarkably well in conserving the mechanical characteristics of the caprine pancreatic tissue. TX-100 has been reported to retain elastic modulus better than SDS in porcine dermis (Joszko et al., 2019). It has also performed well in decellularization of bovine pericardium tissue and retained collagen, sGAG, and Tensile properties better than any other detergent used in the study (Mendoza-Novelo et al., 2011).

### 3.12 Cytocompatibility assay

**FIGURE 12:**
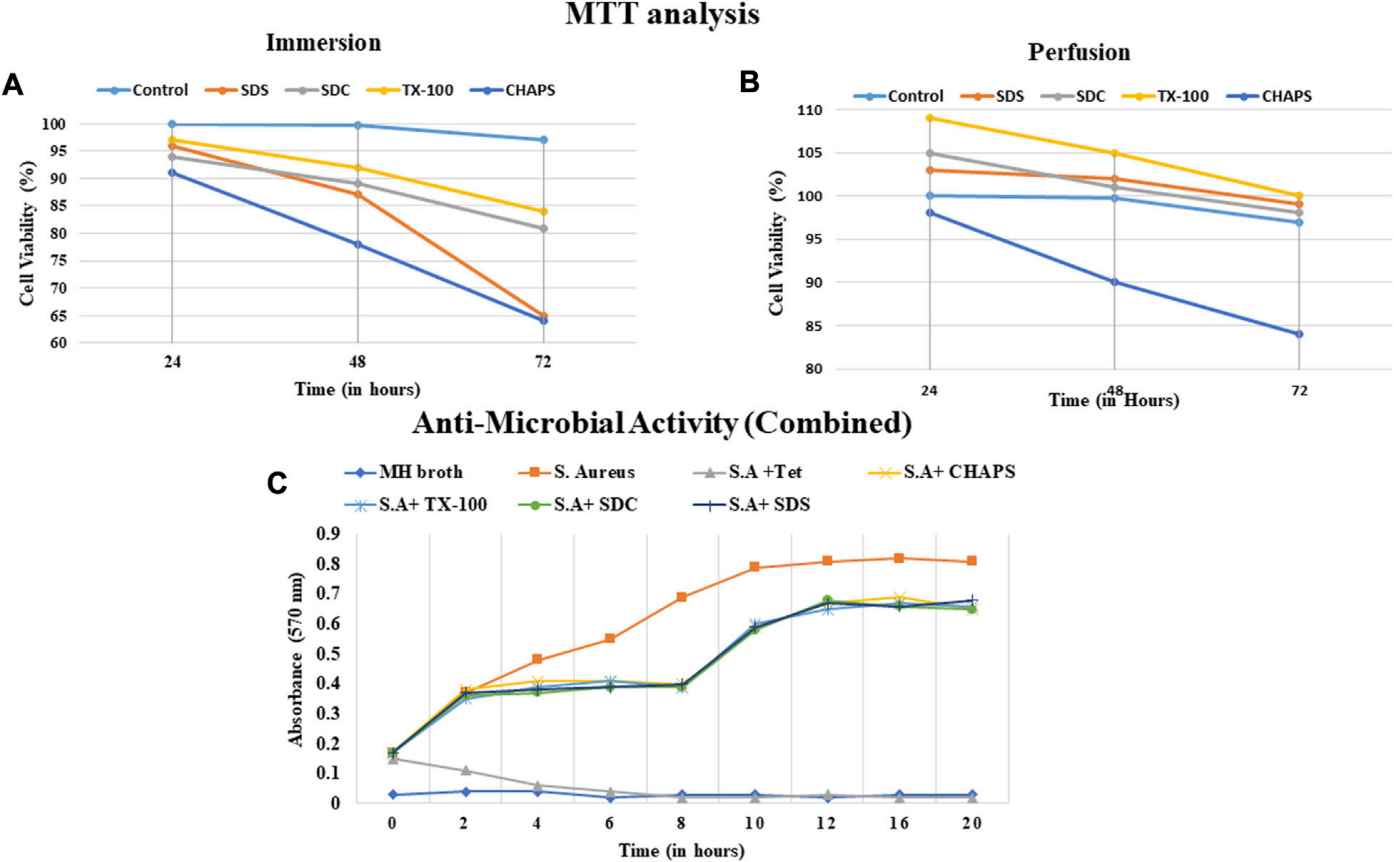
The cell viability with scaffolds obtained via ID **(A)** was unsatisfactory and the CHAPS (a, dark blue) eliminated the cells significantly faster than other detergents. The cell viability% was found to be comparable in both SDC and TX-100 in ID (a, grey and yellow respectively). Overall, the cytocompatibility of PD-treated tissues was found to be increased **(B)**, with the exception of CHAPS (b, dark blue). In PD, TX-100 and SDS have significantly outperformed (b, grey and yellow respectively) the rest of the detergents. The anti-microbial activity of the digested scaffold was detected up to 8 h time stamp **(C)**, but afterwards diminished and led to the increase in the microbial growth.

A biocompatible scaffold will not produce unfavorable responses or damage the cells or tissues that interact with it (Zhou et al., 2022). In case there is an underlying fear of immune response, then masking or cross-linking can also be performed after decellularization to suppress such immune rejection. Masking entails concealing the exposed antigenic motifs on the decellularized tissue scaffold to make it invisible to the recipient’s immune system, hence decreasing the risk of immunological detection and rejection (Chakraborty et al., 2019). Measuring the compatibility of a decellularized scaffold assures that it supports cell attachment, proliferation, and differentiation and promotes tissue regeneration without inducing inflammation, immunological responses, or other negative impacts. In ID, It was observed that CHAPS detergent was the least cytocompatible since it reduced the cell population to 78% within 48 h (Figure 12A); although SDS performed quite well during the initial 48 h, after 72 h, the cell population had fallen by up to 65%, indicating a considerable decline. TX-100 and SDC performed consistently, although TX-100 performed better in this instance and maintained the cell population at 84% for 72 h. In PD, CHAPS did not perform better than other detergents, but it outperformed its ID counterpart. This behavior by CHAPS has been detected in each of the aforementioned experiments, suggesting that the mode and duration of decellularization directly impact the final ECM obtained. The cytocompatibility of the remaining three detergents was found to be superior in PD, with a 98%–100% cell survival rate for each, even after 72 h (Figure 12B). Here, SDS outpaced its ID counterpart (similar to CHAPS) and was discovered to be on the same level as SDC. TX-100 has maintained its outcomes and demonstrated enhanced cell survival efficacy compared to any other detergent in PD. The sudden increase in the number of cells within 24 h could be correlated to the leftover growth factors and cell adhesion receptors imbibed in the intact ECM (Martino et al., 2015). During the incubation phase of the MTT assay, these factors may have contributed to cell growth within 24 h. As evidenced by the H&E staining data, the detergents which resulted in intact ECM (TX-100, SDS, SDC in PD set-up (Figures 4H, J, L), appear to have performed well in MTT also, whereas the detergents that resulted in fragmented ECM in H&E staining (CHAPS, SDS, and SDC in ID set-up, Figures 4E, I, K) have maintained that position by showing lesser cell growth or even rapid death in MTT analysis. This correlation pattern strongly suggests that the components associated with the ECM are responsible for the sharp increase in cell growth within 24 h of incubation. When cells are reseeded into this scaffold, the remnant growth factors and cytokines sequestered in the intact ECM web would spatially cue the cells to multiply and operate as they would have in native tissue (Bongolan et al., 2022). Regarding subsequent successful recellularization, it is vital to retain not only the integrity of the ECM network but also the cell adhesion and proliferation factors in the decellularized scaffold. The MTT analysis provides a solid foundation for potential *in-vivo* immune response studies after scaffold implantation that may be undertaken as an expansion of this study.

### 3.13 Antimicrobial activity

The outcomes of the antibacterial investigation indicated that the naturally occurring peptides resulting from the degradation exhibited efficacy against *S. aureus*. The decellularized matrix of both ID and PD tissues released specific bioactive peptide molecules that significantly curtailed the proliferation of *S. aureus* for up to 8 h (Figure 12C). Beyond this initial 8-h window of antibacterial action, the potency of ECM constituents waned, leading to a resurgence in bacterial growth and propagation. This transient antibacterial effect of the degradation products from DT is believed to offer immediate protection to implanted tissue, bridging the time until both the humoral immune response and host cell inflammatory response come into play (Jiménez-Gastélum et al., 2019). Although the precise mechanism underlying the antibacterial action of peptides produced during the degradation of ECM remains somewhat elusive, existing reports suggest that these antibacterial peptides might either directly interact with the bacterial cell membrane, leading to membrane lysis, or diffuse into the bacterial cell cytoplasm, disrupting protein synthesis (Abdillahi et al., 2018). Notably, the intact DT (when not treated with collagenase digestion) exhibited no antibacterial activity, simply as a natural organ would not possess such a capacity. Thereby, the antibacterial results highlight the potential of digested DT to impede bacterial growth, thus showcasing its viability for applications in tissue engineering. (Note: Since there was almost no difference between the antibacterial activity of ID and PD, a weighted average of each sample was calculated).

## 4 Conclusion

To summarize our findings, we can safely say that for obtaining an ECM scaffold from the caprine pancreas, PD should be the favoured mode. The preferred detergents to obtain a biocompatible 3D scaffold are SDS, TX-100, and SDC. SDC, SDS, and TX-100 successfully preserved all the desirable properties in a scaffold, including major ECM components (collagen, fibronectin, and sGAG), tensile properties, comparable biodegradation, and cytocompatibility. Thus, we strongly recommend using SDS in combination with DNase for the decellularization of this tissue. Each of these three detergents (SDS, SDC, and TX-100) consistently maintained their performance in PD, as seen by the data gathered from each test. It will be helpful if SDC and SDS are used with any kind of protease inhibitor. The chemical nature of each detergent has a unique and distinct impact on the composition and framework of the ECM. Although in our study, it was noticed that the same concentration of CHAPS has performed well in PD but not in ID. Hereby, we establish that, in addition to chemical composition, the duration of exposure to the tissue also plays a prominent role in the retention of scaffoldic features of the final ECM. TX-100 has consistently dominated all other detergents in terms of obtaining ECM architecture without any compromise in elastic modulus. Another notable finding in the PD was the elevated proportions of sGAG and collagen (SDC, SDS, and TX-100) after decellularization, which could be the result of the unmasking of these two ECM components following lesser exposure time to the ECM. Apart from collagen, the cell population in the MTT analysis was also seen to be increased. We have previously established that caprine pancreatic ECM promotes cell migration, adhesion, and proliferation under *in vitro* culture conditions; therefore, this increase in cell population can be attributed to the abundance of ECM-associated molecules persisting in the scaffold (Singh et al., 2022). M1 macrophages can contribute to the initial inflammatory response to such scaffolds, which can lead to tissue damage if not appropriately controlled. M2 macrophages are important for the transition from this initial inflammatory response to a more regenerative and healing environment. In order to limit the M1-mediated pro-inflammatory response while encouraging M2-mediated tissue repair and regeneration *in-vivo*, it is crucial to examine the balance between the responses of M1 and M2 macrophages (Chakraborty et al., 2020). The physical strength of ECM also plays a critical part in characterizing the scaffold as transplantable. The detergents assessed in this study under PD have shown promising results in securing the elasticity of the ECM, except for CHAPS. Here, again, CHAPS exceeded its ID counterpart, consistently indicating that the exposure time of detergent lowers the desired scaffold characteristics. In PD, TX-100 exhibited the most extension at break, whereas CHAPS exhibited the least, indicating that TX-100 DT is capable of undergoing maximum elongation without fracture. This tensile property is advantageous when a scaffold is to be transplanted and will be subjected to usual stresses during handling as well as from the surrounding organs. The scaffold must be willing to withstand these forces and return to its normal shape when they are removed. The findings from the antimicrobial investigation indicate the presence of various antibacterial low-molecular-weight peptides within the ECM, which might contribute to elucidating the mechanism behind the bacterial infection protection offered by these biological scaffolds. Consequently, if the decellularized matrix is employed as a scaffold for tissue engineering, its gradual *in-vivo* degradation would result in the continuous release of antibacterial peptides, affording a sustained antibacterial impact within the host. It was noticeable that there are differences in mechanical properties, degradation, swelling, and cell viability in the perfusion method vs. the immersion method; this can be explained based on the methods employed for these. Perfusion can lead to more thorough and uniform removal of cellular materials in a lesser span of time, giving less time for detergents to interact with ECM. This can result in a more consistent and homogenous scaffold which results in a better architecture and structure of the ECM, which is important for maintaining the biological and mechanical properties of the scaffold. Notably, the pressure and flow rates applied during perfusion plays the most crucial role in protecting the delicate ECM structure. The ECM can be disrupted by excessive pressure and flow, resulting in changes to the aforementioned properties. Moreover, ID may not penetrate thick tissues as efficiently as PD, leaving cellular components within the interior of the tissue and resulting in less homogeneous decellularization compared to PD, particularly in larger and denser tissues. Immersion necessitates more time to decellularize the scaffold, which entails leaving the ECM in close contact with the detergents for a longer period of time, which prolongs cell-ECM interactions, ultimately resulting in scaffolds with inferior mechanical and biological properties. Regarding cell viability, the perfusion method offers better intact vasculature and oxygenation, supporting higher cell viability compared to the immersion method, which may suffer from localized shrinking of vasculature and oxygen deficiency. To sum up our findings, we suggest the use of SDC, SDS, and TX-100 for perfusion decellularization of caprine pancreatic ECM. These detergents can be employed alone or in conjunction with each other to expedite decellularization, as the shortest detergent exposure period yields better-preserved ECM architecture resulting in better physical properties. In addition to detergents, the use of any protease inhibitor or DNase is highly recommended. These conclusions cannot be extended to any other tissue other than caprine pancreatic ECM. Further expansion of this study can be undertaken with the recellularization of this scaffold and its immunological response study *in-vivo* with multiple clinical trials in order to establish it as a novel scaffold source. Consequently, caution should be exercised when translating results from animal models to human contexts, and further research, including human clinical studies, is crucial for comprehensive and reliable insights into human inflammatory responses.

## Data Availability

The raw data supporting the conclusions of this article will be made available by the authors, without undue reservation.

## Abbreviations

CHAPS, (3-((3-cholamidopropyl) dimethylammonio)-1-propanesulfonate); DM, Decellularized matrix; DPX, Dibutylphthalate polystyrene xylene; DT, Decellularized tissue; ECM, Extracellular matrix; GLAA, Glacial acetic acid; ID, Immersion decellularization; IHC, Immunohistochemical; n.s, Not significant; NT, Native Tissue; PBS, Phosphate buffer saline; PD, Perfusion decellularization; resp, respectively; RT, Room temperature; SDC, Sodium deoxycholate; SDS, Sodium dodecyl sulfate; sGAG, Sulphated glycosaminoglycans; TBST, Tris buffer saline with 0.1% Tween 20 (v/v); TX-100, Triton X-100; Y. M, Young’s Modulus.
